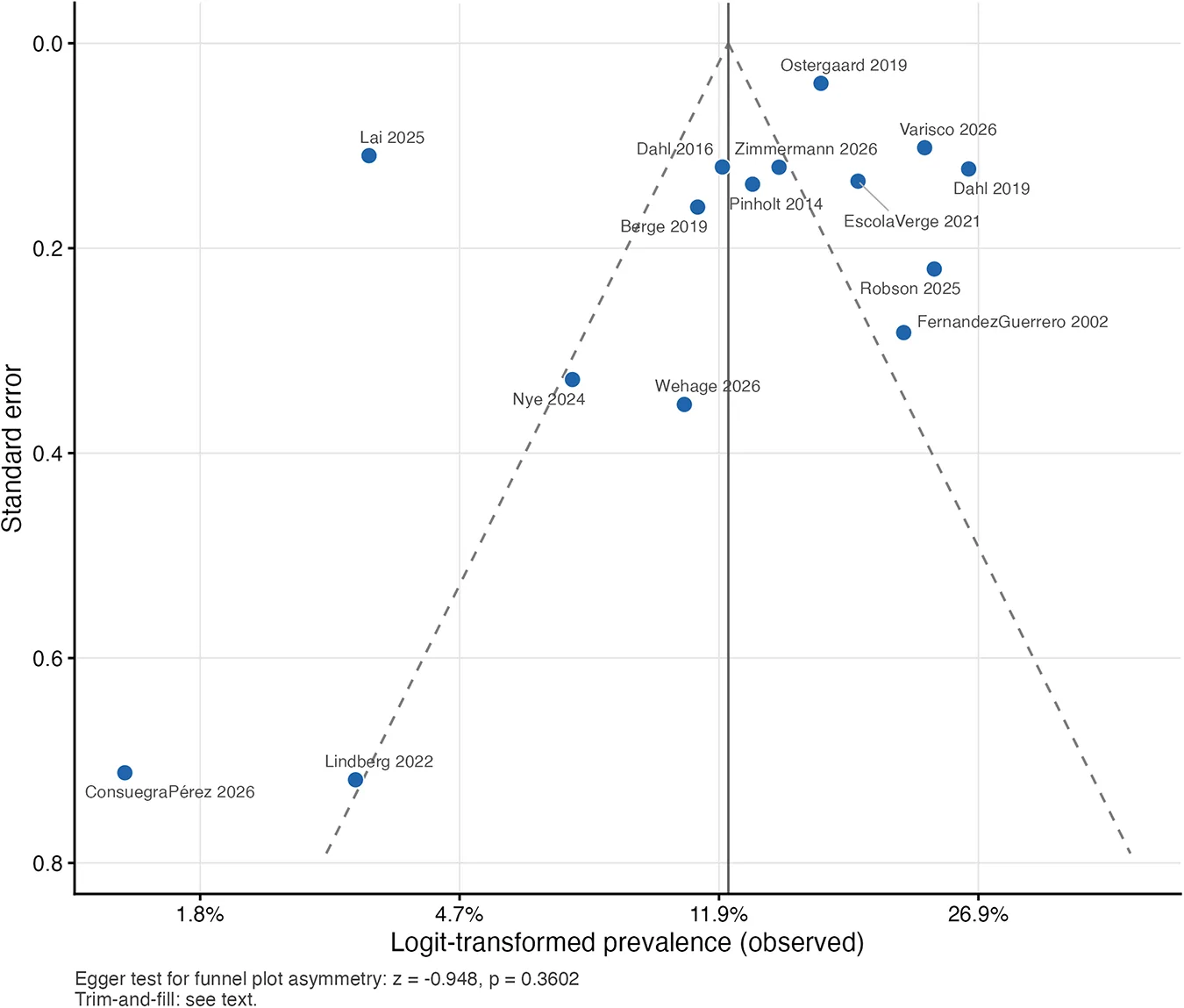# *Enterococcus faecalis* bacteraemia and infective endocarditis: a systematic review and meta-analysis

**DOI:** 10.1016/j.eclinm.2026.104241

**Published:** 2026-09-22

**Authors:** Giovanni Mori, Alessandro Castelli, Andrea Cordin, Giovanni Lorenzin, Roberto Bonmassari, Francesco Onorati, Alberto Maino, Massimiliano Lanzafame, Vito Racanelli, Nicola Susca

**Affiliations:** a Infectious Diseases Unit, Santa Chiara Hospital, Azienda Sanitaria Universitaria Integrata del Trentino (ASUIT), Trento, Italy; b Center for Medical Sciences (CISMed), University of Trento, Trento, Italy; c Microbiology Unit, Santa Chiara Hospital, Azienda Sanitaria Universitaria Integrata del Trentino (ASUIT), Trento, Italy; d Department of Cardiology, Santa Chiara Hospital, Azienda Sanitaria Universitaria Integrata del Trentino (ASUIT), Trento, Italy; e Department of Cardiac Surgery, Santa Chiara Hospital, Azienda Sanitaria Universitaria Integrata del Trentino (ASUIT), Trento, Italy; f Internal Medicine Division, Santa Chiara Hospital, Azienda Sanitaria Universitaria Integrata del Trentino (ASUIT), Trento, Italy

**Keywords:** Infective endocarditis, *Enterococcus faecalis* bacteraemia, Echocardiography, Meta-analysis, Ascertainment bias

## Abstract

**Background:**

Rates of infective endocarditis (IE) in *Enterococcus faecalis* bloodstream infection (EF-BSI) vary more than 20-fold across published cohorts. We aimed to estimate the true IE prevalence, quantify the role of echocardiographic ascertainment in driving this variation, and assess the diagnostic performance of IE prediction scores.

**Methods:**

We conducted a systematic review and meta-analysis (PubMed, Embase, Scopus, and Web of Science searched from inception to 16 August 2026; PROSPERO registration CRD420251240242) of observational studies reporting IE prevalence in confirmed EF-BSI episodes. Risk of bias was assessed with the Quality in Prognostic Studies (QUIPS) tool. Pooled prevalence was estimated with a generalised linear mixed model (logit scale). Meta-regression with echocardiographic coverage as the moderator was used to derive an ascertainment-adjusted prevalence. Diagnostic performance of the NOVA, DENOVA, and MEFIER scores was summarised by random-effects pooling.

**Findings:**

Fifteen studies (11,157 EF-BSI episodes) were included. Crude pooled IE prevalence was 11.8% (95% confidence interval [CI] 8.1–16.8; I^2^ = 97.3%). In studies with systematic echocardiography (k = 2), pooled prevalence was 24.2% (21.5–27.2; I^2^ = 0.0%). Meta-regression explained up to 91.5% of between-study variance; estimated plateau IE prevalence was approximately 30%. Pooled DENOVA sensitivity was 88.7% (78.9–94.3%) and specificity 84.0% (77.8–88.7%); prospective validation yielded sensitivity 79.2% (negative predictive value [NPV] 93.0%). NOVA had high sensitivity (95.6%) but low specificity (30.7%). Certainty of evidence was low (Grading of Recommendations Assessment, Development and Evaluation [GRADE]).

**Interpretation:**

Once ascertainment is accounted for, approximately one quarter of EF-BSI episodes are complicated by IE. These findings support a shift from selective to routine echocardiography, with transoesophageal echocardiography (TEE) as the definitive modality. Existing scores can prioritise and sequence imaging but do not identify a subgroup in whom it may be safely omitted.

**Funding:**

10.13039/501100009629Cassa di Risparmio di Trento e Rovereto (GRAM+ TN project).

**Research in context**

**Evidence before this study**

We searched PubMed, Embase, Scopus, and Web of Science (inception to August 16, 2026) using terms for *Enterococcus faecalis*, bacteraemia, and infective endocarditis (IE), without language restriction. No prior systematic review or meta-analysis was identified. Published cohorts reported widely discordant IE rates in *E. faecalis* bloodstream infection (EF-BSI) (1–26%), with the highest estimates from prospective studies applying systematic echocardiography to all patients and the lowest from retrospective cohorts with selective imaging. Most studies were at moderate to high risk of bias and used heterogeneous diagnostic criteria. Three prediction scores (NOVA, DENOVA, and MEFIER) had been developed to risk-stratify patients and compared within individual cohorts, but no systematic synthesis with pooled diagnostic accuracy estimates across independent studies was available. Whether the variation in reported prevalence reflected genuine epidemiological differences or differential echocardiographic ascertainment had not been formally tested, and no study had evaluated this question within the framework of the 2023 Duke–International Society for Cardiovascular Infectious Diseases (ISCVID) criteria, which reclassify *E. faecalis* as a typical IE organism.

**Added value of this study**

To our knowledge, this is the first systematic review and meta-analysis of IE prevalence in EF-BSI. Most between-study heterogeneity was explained by echocardiographic ascertainment intensity: a quadratic meta-regression with coverage as the moderator explained up to 91.5% of between-study variance, yielding an ascertainment-adjusted plateau prevalence of approximately 30%, consistent with the prevalence observed directly in screen-all cohorts. As far as we are aware, this study also provides the first pooled diagnostic accuracy estimates for NOVA and DENOVA across independent external validation studies, including the largest prospective cohort to date.

**Implications of all the available evidence**

The true IE burden in EF-BSI is substantially higher than routine practice figures suggest, supporting a shift from selective to routine echocardiography. The optimal sequence and modality (transthoracic echocardiography [TTE] first, with transoesophageal echocardiography [TEE] reserved for high-risk or inconclusive cases, or direct TEE in all) requires prospective, outcome-based evaluation. Existing prediction scores can prioritise and sequence imaging but cannot safely define a subgroup in whom it may be omitted. A prediction rule recalibrated to the Duke–ISCVID 2023 criteria and prospectively validated against protocol echocardiography is an immediate research priority.

## Introduction

*Enterococcus faecalis* (EF) bacteraemia carries a paradox at its core: reported rates of infective endocarditis (IE) range from 1% to 26.1% across published cohorts, a more than 20-fold variation that has shaped clinical practice for decades.^1^, ^2^, ^3^, ^4^, ^5^ EF is a major cause of bloodstream infection (BSI) in older adults, carries substantial short-term mortality in large cohort studies,^1^^,^^6^ and is one of the leading microbiological causes of IE.^7^ In a landmark prospective study with systematic echocardiography, including transoesophageal echocardiography (TEE) in 74% of patients, definite IE was diagnosed in 26.1% of EF-BSI episodes.^2^ However, markedly lower estimates have been observed in retrospective cohorts with less systematic cardiac imaging, raising the possibility that a considerable proportion of EF-IE remains unrecognised in routine practice.^1^^,^^3^, ^4^, ^5^ Echocardiography is therefore both the diagnostic gold standard for IE and a major source of detection bias in prevalence estimates: whether the lower figures from routine practice reflect a genuinely lower disease burden or simply under-ascertainment has never been formally tested. Current European guidelines support a low threshold for echocardiographic evaluation in patients with EF-BSI.^8^ This position is reinforced by the 2023 Duke–International Society for Cardiovascular Infectious Diseases (ISCVID) criteria, which classify EF as a typical IE microorganism regardless of source or acquisition setting, thereby strengthening the diagnostic relevance of EF-BSI in the evaluation of suspected IE.^9^ To support risk-adapted echocardiography, several clinical prediction tools have been developed, including NOVA,^5^ DENOVA,^10^ and MEFIER.^11^ Although these scores have shown promising discrimination, they were derived or validated in geographically restricted cohorts, under heterogeneous echocardiographic strategies, and largely outside a contemporary Duke–ISCVID 2023 framework. Consequently, their comparative performance, generalisability, and implications for the risk of missed EF-IE remain uncertain.

We therefore conducted a systematic review and meta-analysis to test whether between-study heterogeneity in EF-IE prevalence reflects echocardiographic ascertainment intensity, to estimate the prevalence of IE among EF-BSI episodes with ascertainment adjustment, to characterise clinical predictors of IE, and to summarise the diagnostic performance of existing prediction scores.

## Methods

This systematic review and meta-analysis was conducted in accordance with the PRISMA 2020 statement^12^ and prospectively registered in the International Prospective Register of Systematic Reviews (PROSPERO; CRD420251240242). Full methodological details are provided in the Supplementary Methods.

### Eligibility criteria

Observational studies reporting the prevalence of IE in patients with microbiologically confirmed EF-BSI were eligible, irrespective of design or primary objective. Excluded were studies of mixed bacterial populations in which EF-BSI episodes could not be separated, conference abstracts, case–control designs, IE-enriched denominators, secondary reanalyses, and overlapping cohorts. Studies using non-standard diagnostic criteria (e.g., International Classification of Diseases, 10th revision [ICD-10] coding) or without systematic echocardiography were retained and addressed in stratified or sensitivity analyses.

### Literature search and data extraction

PubMed, Scopus, Web of Science, and Embase were searched from inception to 20 January 2026 (full search string in Supplementary Methods), and the search was subsequently re-run on 16 August 2026 using the identical strategy to capture records added after the primary search. Rayyan (rayyan.ai) was used to facilitate title and abstract screening. Two reviewers (G.M. and N.S.) independently screened records; disagreements were resolved by a third (Al.Ca.). Data were extracted on study characteristics, outcomes, IE predictors, prediction score performance, and echocardiographic strategy. Risk of bias (RoB) was assessed with the Quality in Prognostic Studies (QUIPS) tool.^13^

### Statistical analysis

Pooled IE prevalence was estimated with a generalised linear mixed model (GLMM) with a logit link, which models the within-study binomial distribution of IE cases directly and represents between-study variation through a study-level random effect, fitted by maximum likelihood (ML); pooled estimates and 95% confidence intervals (CI) were back-transformed to the prevalence scale. Heterogeneity was quantified by I^2^ and between-study variance by τ^2^. Meta-regression with TEE and transthoracic echocardiography (TTE) coverage as separate moderators (linear and quadratic models) was used to estimate ascertainment-adjusted IE prevalence; restricted maximum likelihood (REML) was used for coefficient estimation and R^2^ (defined as the proportional reduction in between-study variance relative to the intercept-only model); models were refitted with ML for comparison by the Akaike Information Criterion (AIC) and the Likelihood Ratio Test (LRT), because REML log-likelihoods are not comparable across models with different fixed-effect structures. Where the quadratic coefficient was negative, the vertex of a concave-down quadratic curve was interpreted as the plateau prevalence under near-complete echocardiographic coverage and back-transformed for presentation. Leave-one-out sensitivity analyses assessed model stability. All analyses were repeated substituting modified Duke 2000 criteria for the Varisco et al. study.^14^ Diagnostic performance of NOVA, DENOVA, and MEFIER was summarised as sensitivity, specificity, and area under the receiver operating characteristic curve (AUROC); sensitivity and specificity were pooled as logit-transformed proportions with random-effects models; AUROC values were pooled on the logit-scale with within-study variances derived from the reported CI Publication bias was assessed by funnel plot inspection, Egger's test, and the trim-and-fill method. Evidence certainty was rated with the Grading of Recommendations Assessment, Development and Evaluation (GRADE) framework across domains of risk of bias, inconsistency, indirectness, imprecision, and publication bias. A two-sided p < 0.05 was considered statistically significant.

### Role of the funding source

This study received no external funding. The corresponding author had full access to all data and had final responsibility for the decision to submit for publication.

## Results

### Studies’ selection

Fig. 1 shows the PRISMA flow diagram. The search of the four databases retrieved 2265 records; after duplicate removal, 1274 unique records were screened by title and abstract. Of these, 1217 were excluded as irrelevant and 57 were assessed at full text. Two further articles were identified from the authors' personal files and from citation screening. Of these 59 reports, 44 were excluded (21 for mixed populations or non-separable EF-BSI episodes, 10 conference abstracts, 10 incompatible designs, and 3 overlapping cohorts), leaving 15 studies for the meta-analysis.^1^, ^2^, ^3^, ^4^^,^^10^^,^^11^^,^^14^, ^15^, ^16^, ^17^, ^18^, ^19^, ^20^, ^21^, ^22^

**Fig. 1:**
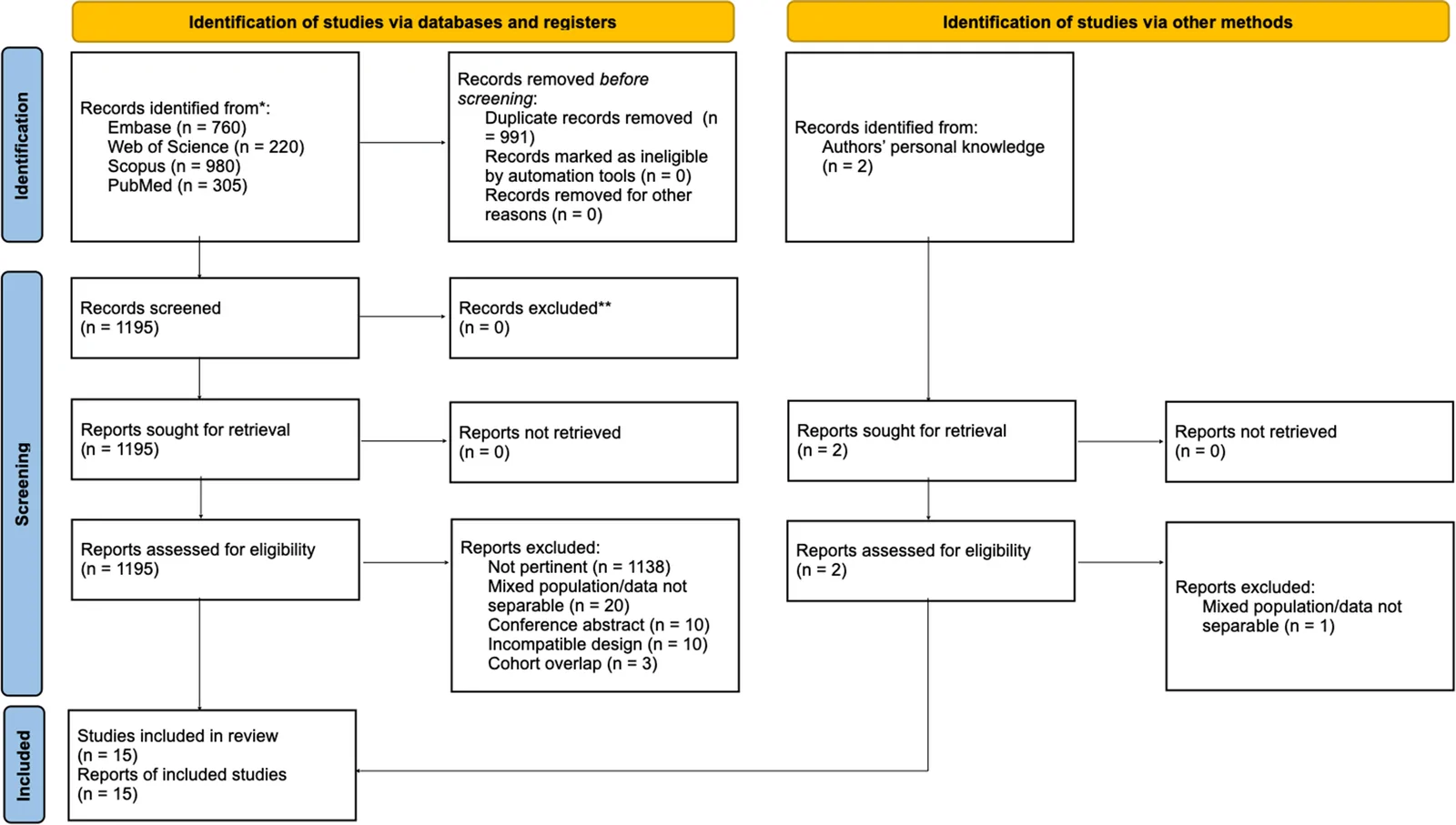
**Preferred Reporting Items for Systematic Reviews and Meta-Analyses (PRISMA) 2020 flow diagram of study selection**.

### Characteristics of the included studies and RoB assessment

Table 1 shows the characteristics and RoB assessment of the included studies. Three were prospective and 12 retrospective, with enrolment spanning 1995 to 2024. In total, 11,157 EF-BSI episodes were analysed, including 1483 IE cases, and the observed IE prevalence ranged from 1.0% to 26.1%. IE was defined by the modified Duke 2000 criteria in four studies, the Duke–ISCVID 2023 criteria in three, ICD-10 codes in two, the original 1994 Duke criteria in one, the modified European Society of Cardiology (ESC) 2015 criteria in one, and the ESC 2023 criteria in one; two studies applied both the modified Duke 2000 and ESC 2015 criteria, and one did not report the criteria used. Echocardiography followed a systematic strategy in two studies and a selective strategy in 12, while one registry-based study did not report its strategy. The two systematic-imaging studies performed TTE in all patients and TEE in 74% and 30.2%, respectively. Eight studies reported the diagnostic performance of NOVA, DENOVA, or MEFIER. Overall risk of bias was low in two studies, moderate in six, and high in seven (the full RoB assessment is reported in Supplementary Table S1).

**Table 1:** Characteristics of included studies.

Study	Country	Study design	Enrolment	N. EF-BSI	N. IE	IE prevalence (95% CI)	IE criteria	Echo strategy	Echo coverage (TTE/TEE)	Prediction scores	RoB (overall)
Fernandez-Guerrero 2002	Spain	Prospective cohort	1995–1999	74	16	21.6% (13.8%–32.3%)	Duke criteria (original 1994, Durack et al.)	Selective	NR/NR	None	High
Pinholt 2014	Denmark	Retrospective cohort	2006–2009	457	61	13.3% (10.5%–16.8%)	Modified Duke criteria (Li 2000) — definite + possible IE	Selective	NR/NR	None	Moderate
Dahl 2016	Denmark	Retrospective cohort	2010–2013	647	78	12.1% (9.8%–14.8%)	Modified Duke criteria (Li 2000)	Selective	36.3%/17.9%	NOVA	Moderate
Berge 2019	Sweden	Retrospective cohort	2012–2016	397	44	11.1% (8.4%–14.6%)	Modified Duke criteria (Li 2000) — definite IE	Selective	29.2%/21.7%	DENOVA, NOVA	Moderate
Dahl 2019	Denmark	Prospective cohort	2014–2016	344	90	26.2% (21.8%–31.1%)	Modified Duke + ESC 2015 criteria	Systematic	100.0%/74.0%	None	Low
Ostergaard 2019	Denmark	Retrospective cohort	2010–2017	4700	785	16.7% (15.7%–17.8%)	ICD-10 codes — PPV validated at 90%	NR	NR/NR	None	High
Escola Verge 2021	Spain	Retrospective cohort + Prospective registry	2007–2018	362	68	18.8% (15.1%–23.1%)	Modified Duke criteria (Li 2000) until 2015; ESC 2015 modified criteria from 2015 onward.	Selective	39.0%/30.9%	None	Moderate
Lindberg 2022	Sweden	Retrospective cohort	2017–2019	62	2	3.2% (0.9%–11.0%)	ESC 2015 modified criteria	Selective	19.4%/21.0%	DENOVA, NOVA	High
Nye 2024	United Kingdom	Retrospective cohort	2017–2022	140	10	7.1% (3.9%–12.6%)	Not reported	Selective	37.9%/2.9%	None	High
Lai 2025	China	Retrospective cohort	2009–2019	2535	86	3.4% (2.8%–4.2%)	ICD-10 codes — PPV not validated	Selective	NR/NR	MEFIER	High
Robson 2025	Australia	Retrospective cohort	2017–2021	114	27	23.7% (16.8%–32.3%)	Duke-ISCVID 2023	Selective	72.8%/27.2%	NA	Moderate
Consuegra Pérez 2026	Spain	Retrospective cohort	2014–2023	148	2	1.4% (0.4%–4.8%)	ESC 2023 criteria (Delgado et al., Eur Heart J 2023)	Selective	14.9%/4.7%	DENOVA, NOVA	High
Varisco 2026	Multinational	Prospective multicentre cohort	2019–2024	543	125	23.0% (19.7%–26.7%)	Duke-ISCVID 2023 (Modified Duke 2000 also available: 72/543 = 13.3%)	Systematic	100.0%/30.2%	DENOVA, NOVA	Low
Wehage 2026	Germany	Retrospective cohort	2021–2022	85	9	10.6% (5.7%–18.9%)	Modified Duke criteria (Li 2000)	Selective	36.5%/18.8%	DENOVA	High
Zimmermann 2026	Switzerland	Retrospective cohort	2015–2024	549	80	14.6% (11.9%–17.8%)	Duke-ISCVID 2023 (definite)	Selective	NR/NR	DENOVA, MEFIER, NOVA	Moderate

IE prevalence was calculated as definite IE cases/total EF-BSI episodes; 95% CIs were estimated using the Wilson score method. Echo coverage is expressed as the proportion of patients undergoing any echocardiography and TEE, respectively. Risk of bias was assessed with the Quality in Prognostic Studies (QUIPS) tool across six domains: study participation, attrition, prognostic factor measurement, outcome measurement, confounding, and statistical analysis; each domain and the overall study were rated as low, moderate, or high risk of bias. EF-BSI = *Enterococcus faecalis* bloodstream infection; IE = infective endocarditis; TTE = transthoracic echocardiography; TEE = transoesophageal echocardiography; ESC = European Society of Cardiology; NR = not reported; PPV = positive predictive value; RoB = risk of bias.

### Pooled prevalence of IE in EF-BSI

The pooled prevalence of IE in EF-BSI was 11.8% (95% CI 8.1–16.8) (Fig. 2). Heterogeneity was substantial (I^2^ 97.3%, τ^2^ 0.5949), reflecting variation in IE diagnostic criteria, geographical area, study period, patient population, and, most notably, echocardiographic coverage. A sensitivity analysis substituting modified Duke 2000 criteria for the Varisco et al. study^14^ is shown in Supplementary Figure S1 (pooled 11.3%, 95% CI 7.9–16.0; I^2^ = 96.9%, τ^2^ = 0.5404). The pooled IE prevalence was higher in studies with systematic echocardiography (24.2%, 95% CI 21.5–27.2; I^2^ = 0%, τ^2^ = 0) and lower in studies with selective echocardiography (9.9%, 95% CI 6.4–14.8; I^2^ = 95.1%, τ^2^ = 0.5841) (Supplementary Figure S2). Geographic stratification was of limited informative value given European predominance (13 of 15 studies); the pooled European prevalence was 12.5% (8.9–17.4%), with high heterogeneity (Supplementary Figure S3). When stratified by median year of enrolment, the pooled prevalence was 14.5% (95% CI 11.8–17.8; I^2^ = 0%) before 2010, 10.6% (6.2–17.5; I^2^ = 98.3%) in 2010–2019, and 12.8% (6.7–22.9; I^2^ = 82.7%) in 2020 and after (Supplementary Figure S4). Stratification by diagnostic criteria yielded a pooled prevalence of 14.9% (95% CI 11.3–19.5%; I^2^ = 86.0%) within the modified Duke 2000 subset, 19.7% (15.0–25.3%; I^2^ = 76.2%) in the Duke–ISCVID 2023 subset, and 7.8% (2.4–22.0%; I^2^ = 99.1%) in the ICD-10 subset (Supplementary Figure S5).

**Fig. 2:**
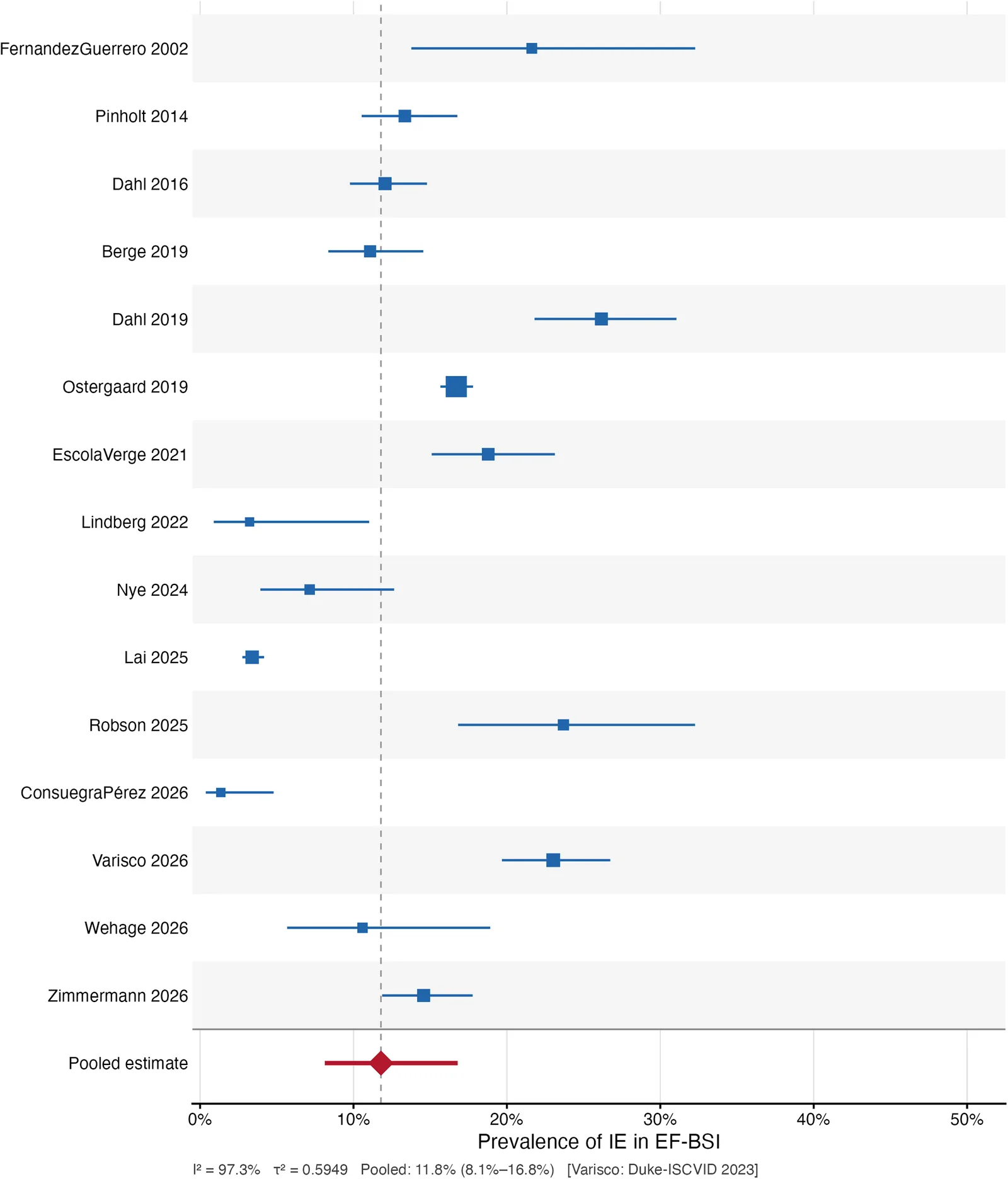
**Forest plot of the pooled prevalence of infective endocarditis (IE) in Enterococcus faecalis bloodstream infection (EF-BSI).** Squares represent individual study estimates; horizontal lines, 95% confidence intervals; square size is proportional to study weight. The diamond represents the random-effects pooled estimate (logit scale). IE was defined according to the Duke–ISCVID 2023 criteria for the Varisco et al. (2026) study; all other studies applied previously existing criteria (see Table 1). I^2^ = between-study heterogeneity statistic; τ^2^ = estimated between-study variance.

The stratified analysis between studies involving tertiary referral centres and registry-based or mixed studies showed identical IE prevalence between the two groups [11.8% (95% CI 6.6%–20.1%) vs 11.8% (95% CI 7.2%–18.7%)], with an R^2^ = 0% and residual I^2^ = 97.1%. Moreover, the two studies with the highest echocardiographic coverage and the lowest risk of bias report similar IE prevalence values, despite being in the tertiary- and non-tertiary-centre groups, respectively (Supplementary Figure S6).

The analysis on IE prevalence conducted excluding high-risk-of-bias studies yielded a pooled prevalence of 17.0% (13.6–21.1%; I^2^ = 87.6%) (Supplementary Figure S7) (Supplementary Table S2). The exclusion of the 11 studies with echocardiographic coverage below 50% showed an IE prevalence of 21.3% (16.8–26.5%; I^2^ = 79.9%) (Supplementary Table S2), and the stratified analysis between overall high, moderate, and low RoB showed a monotonically increasing IE prevalence (Supplementary Table S2).

### Meta-regression

Meta-regression assessed whether echocardiographic coverage explained between-study variance. Fig. 3A shows the linear and quadratic models with TEE as the moderator. The quadratic model (R^2^ = 91.5%) was preferred over the linear (R^2^ = 57.8%) by AIC (17.0 vs 24.4) and LRT (p = 0.002). The quadratic coefficient was negative (Table 2), yielding an estimated plateau IE prevalence of 30.4% (95% CI 23.1–38.9%) at TEE coverage of 58.0% (95% CI 47.0–69.0%); residual heterogeneity was 58.0%. Fig. 3B shows the same analysis for TTE coverage; the quadratic model (R^2^ = 87.4%) was again preferred (AIC 14.3 vs 20.0; LRT p = 0.006), with an estimated plateau of 29.4% (95% CI 20.8–39.8%) at TTE coverage of 79.0% (95% CI 67.0–91.0%); residual heterogeneity was 67.3%. The modified Duke 2000 sensitivity analysis yielded broadly consistent results (Supplementary Figure S8 and Supplementary Table S3). Leave-one-out analyses confirmed the stability of both quadratic models; the only exception was removal of Dahl et al.,^2^ the sole study with TEE coverage above the estimated vertex, which reversed the sign of the quadratic coefficient (Supplementary Table S4). The estimated plateau prevalence from both models was similar to the pooled estimate from the systematic-imaging subgroup.

**Fig. 3:**
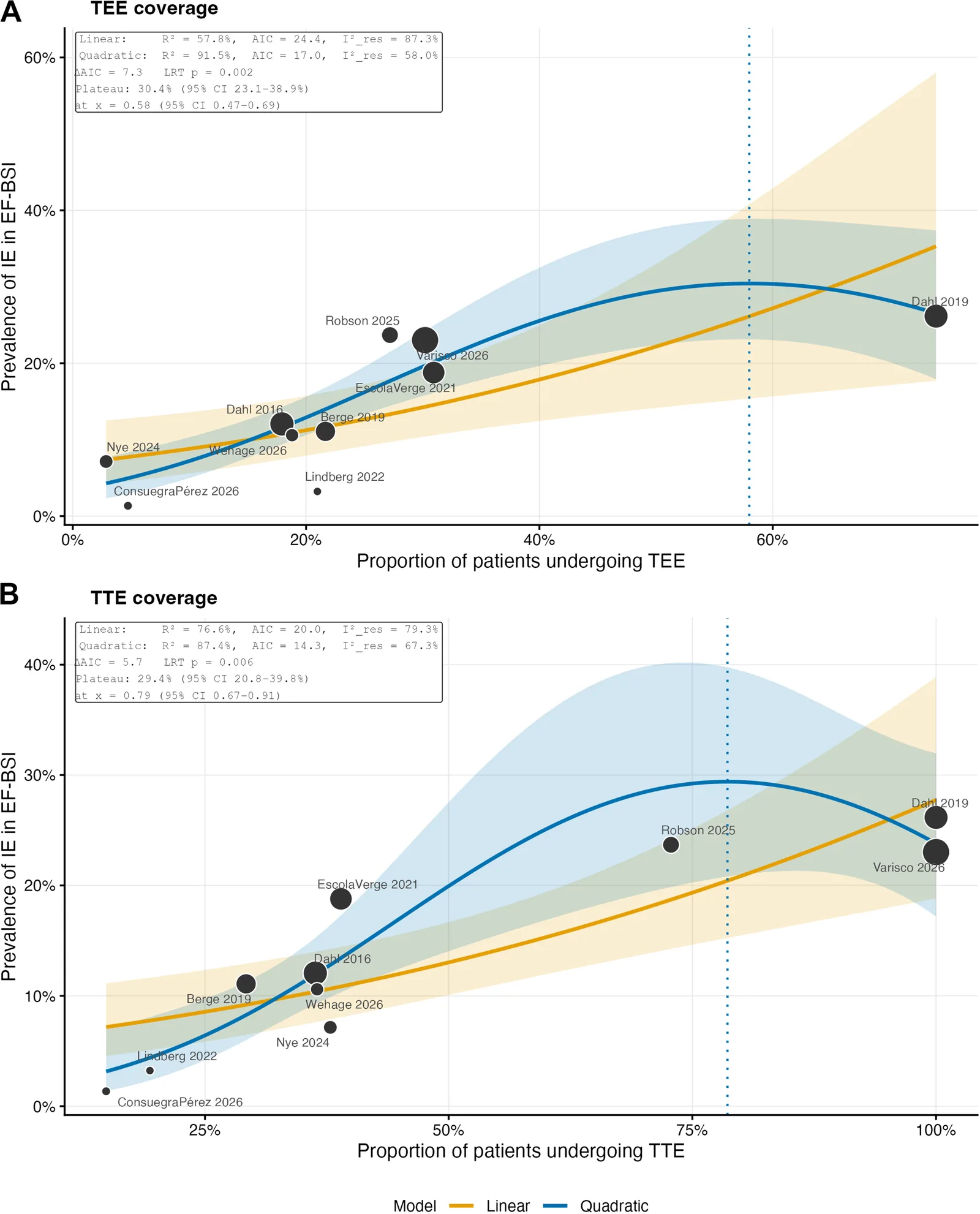
**Meta-regression of IE prevalence in EF-BSI as a function of echocardiographic coverage.** Each circle represents one study; size is proportional to study weight. Orange and blue lines show the fitted linear and quadratic models, respectively; shaded bands indicate 95% confidence intervals. The vertical dashed line marks the estimated plateau (vertex of the quadratic model). **(A)** TEE as moderator: quadratic model R^2^ = 91.5%, AIC = 17.0 (vs 24.4 linear; ΔAIC = 7.4; LRT p = 0.002); residual I^2^ = 58.0%; estimated plateau 30.4% (95% CI 23.1–38.9%) at TEE coverage 58% (95% CI 47–69%). **(B)** TTE as moderator: quadratic model R^2^ = 87.4%, AIC = 14.3 (vs 20.0 linear; ΔAIC = 5.7; LRT p = 0.006); residual I^2^ = 67.3%; estimated plateau 29.4% (95% CI 20.8–39.8%) at TTE coverage 79% (95% CI 67–91%). IE = infective endocarditis; EF-BSI = *Enterococcus faecalis* bloodstream infection; TEE = transoesophageal echocardiography; TTE = transthoracic echocardiography; AIC = Akaike information criterion; LRT = likelihood ratio test.

**Table 2:** Meta-regression model coefficients for the association between echocardiographic coverage and IE prevalence in EF-BSI.

Moderator	Model	Term	Estimate	SE	z	p value	tau^2^	R^2^	AIC
TEE coverage	Linear	Intercept	−2.60774	0.32080	−8.129	<0.001	0.25499	57.8%	24.40
TEE coverage	Linear	TEE proportion	2.70498	0.94717	2.856	0.004	0.25499	57.8%	24.40
TEE coverage	Quadratic	Intercept	−3.34969	0.37410	−8.954	<0.001	0.05112	91.5%	17.00
TEE coverage	Quadratic	TEE proportion	8.70104	2.17475	4.001	<0.001	0.05112	91.5%	17.00
TEE coverage	Quadratic	TEE proportion^2^	−7.50228	2.42533	−3.093	0.002	0.05112	91.5%	17.00
TTE coverage	Linear	Intercept	−2.84001	0.30764	−9.231	<0.001	0.14158	76.6%	20.00
TTE coverage	Linear	TTE proportion	1.88302	0.48462	3.886	<0.001	0.14158	76.6%	20.00
TTE coverage	Quadratic	Intercept	−4.75640	0.83412	−5.702	<0.001	0.07621	87.4%	14.30
TTE coverage	Quadratic	TTE proportion	9.87346	3.19579	3.090	0.002	0.07621	87.4%	14.30
TTE coverage	Quadratic	TTE proportion^2^	−6.28072	2.44214	−2.572	0.010	0.07621	87.4%	14.30

Models were fitted on logit-transformed prevalence using a random-effects meta-regression with the REML estimator. Coefficients are on the logit scale. τ^2^ = residual between-study variance; R^2^ = proportion of total between-study variance explained by the moderator; AIC = Akaike information criterion. EF-BSI = *Enterococcus faecalis* bloodstream infection; IE = infective endocarditis; TEE = transoesophageal echocardiography; TTE = transthoracic echocardiography; SE = standard error.

### Prediction scores diagnostic performance

Seven studies reported the diagnostic performance of the DENOVA score (Fig. 4 and Table 3). The pooled sensitivity was 88.7% (95% CI 78.9–94.3; I^2^ = 50.6%, τ^2^ = 0.3529), pooled specificity 84.0% (77.8–88.7; I^2^ = 84.4%), and pooled AUROC 91.9% (81.0–96.8; I^2^ = 95.9%). Leave-one-out analysis showed that no single estimate determined the pooled result: *Varisco* et al. reported the lowest sensitivity for the DENOVA score while carrying the greatest weight (35.9%); omitting it raised pooled sensitivity to 91.2%, still well below complete sensitivity (Supplementary Table S5). However, it represented the principal source of heterogeneity (LOO I^2^ 0% after its removal). Conversely, the apparent 100% sensitivity in the smaller cohorts should not be read as absolute: Consuegra Pérez and Lindberg each contributed only two endocarditis episodes, with correspondingly wide confidence intervals. The lower sensitivity in Varisco et al. may reflect its use of the Duke-ISCVID 2023 criteria, against which DENOVA was not derived, whereas the earlier validations applied modified Duke 2000, ESC 2015, or ESC 2023 definitions. Seven studies assessed NOVA (Fig. 4 and Table 3): pooled sensitivity 95.6% (88.3–98.4; I^2^ = 56.1%), pooled specificity 30.7% (21.3–42.1; I^2^ = 94.9%), pooled AUROC 86.3% (69.0–94.7; I^2^ = 96.7%). Two studies assessed MEFIER: pooled sensitivity 54.5% (24.9–81.3%; I^2^ = 84.6%), specificity 81.0% (66.2–90.2%; I^2^ = 95.1%), AUROC 79.0% (72.0–88.0%).

**Fig. 4:**
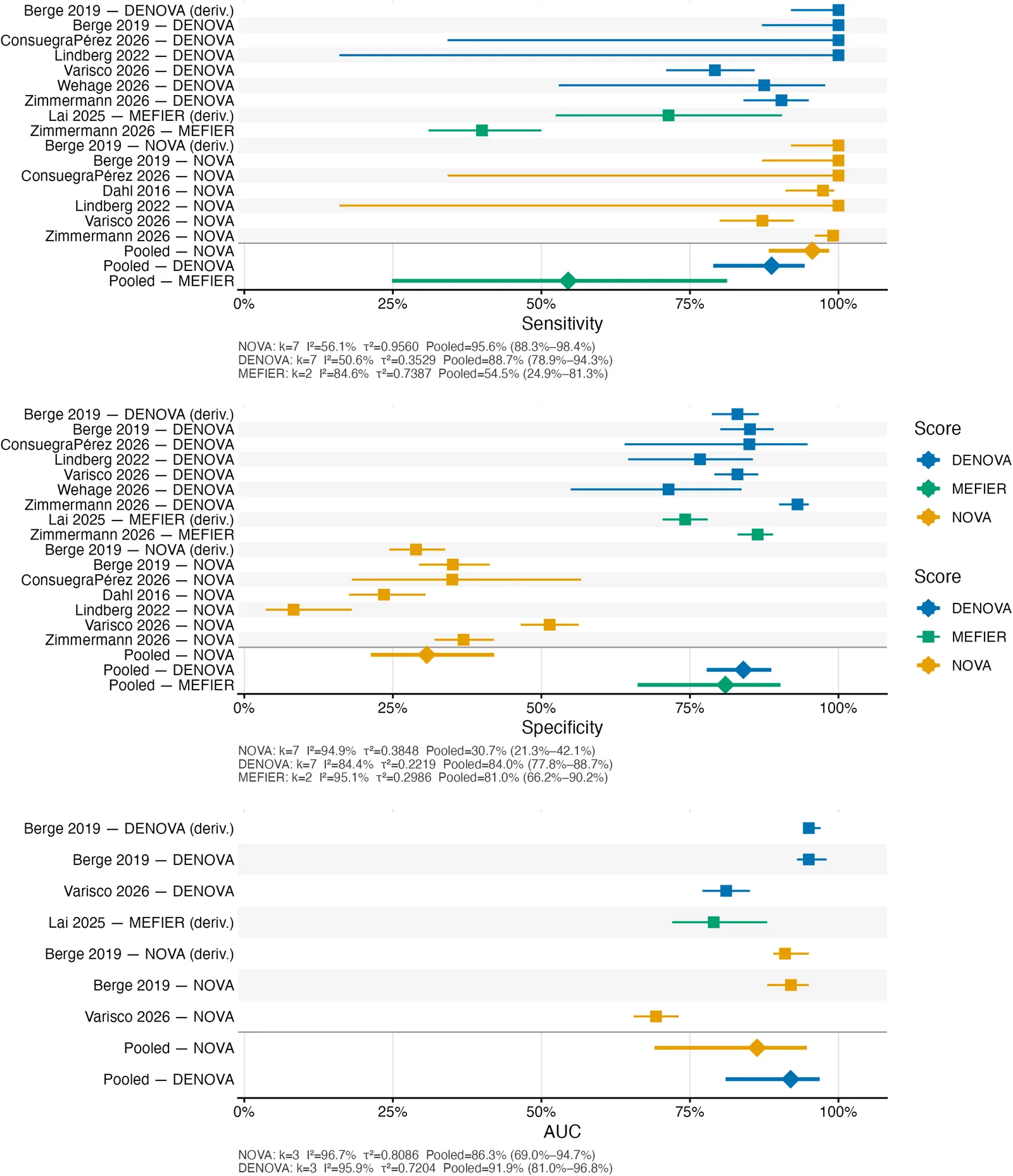
**Performance of the prediction scores for IE in EF-BSI.** Forest plots show sensitivity (top), specificity (middle), and area under the receiver-operating characteristic curve (AUC; bottom). Squares represent individual study estimates with 95% confidence intervals; diamonds represent the random-effects pooled estimate for NOVA, DENOVA and MEFIER. Studies labelled “(deriv.)” are derivation cohorts. Score thresholds applied: NOVA ≥4, DENOVA ≥3, MEFIER as per original publication. IE = infective endocarditis; EF-BSI = *Enterococcus faecalis* bloodstream infection; I^2^ = between-study heterogeneity statistic; τ^2^ = estimated between-study variance; AUC = area under the receiver-operating characteristic curve.

**Table 3:** Diagnostic performance of the prediction scores for IE in EF-BSI.

Study	Score	Type	Threshold	Sensitivity	Specificity	AUC	PPV	NPV
Berge 2019	DENOVA	Derivation	≥3	100.0% (92.0%–100.0%)	83.0% (78.7%–86.6%)	0.950 (0.940–0.970)	42.3%	100.0%
Berge 2019	DENOVA	Validation	≥3	100.0% (87.1%–100.0%)	85.1% (80.1%–89.1%)	0.950 (0.930–0.980)	41.9%	100.0%
Consuegra-Pérez 2026	DENOVA	Validation	≥3	100.0% (34.2%–100.0%)	85.0% (64.0%–94.8%)	NA	40.0%	100.0%
Lindberg 2022	DENOVA	Validation	≥3	100.0% (16.0%–100.0%)	76.7% (64.6%–85.6%)	NA	12.5%	100.0%
Varisco 2026	DENOVA	Validation	≥3	79.2% (71.0%–85.9%)	83.0% (79.1%–86.5%)	0.811 (0.771–0.851)	58.2%	93.0%
Wehage 2026	DENOVA	Validation	≥3	87.5% (52.9%–97.8%)	71.4% (54.9%–83.7%)	NA	41.2%	96.2%
Zimmermann 2026	DENOVA	Validation	≥3	90.4% (84.0%–95.0%)	93.1% (90.0%–95.0%)	NA	78.0%	97.0%
Lai 2025	MEFIER	Derivation	≥32	71.4% (52.4%–90.5%)	74.2% (70.4%–78.0%)	0.790 (0.720–0.880)	11.0%	98.0%
Zimmermann 2026	MEFIER	Validation	≥32	40.0% (31.0%–50.0%)	86.4% (83.0%–89.0%)	NA	44.0%	84.0%
Berge 2019	NOVA	Derivation	≥4	100.0% (92.0%–100.0%)	28.9% (24.4%–33.8%)	0.910 (0.890–0.950)	14.9%	100.0%
Berge 2019	NOVA	Validation	≥4	100.0% (87.1%–100.0%)	35.1% (29.4%–41.3%)	0.920 (0.880–0.950)	14.2%	100.0%
Consuegra-Pérez 2026	NOVA	Validation	≥4	100.0% (34.2%–100.0%)	35.0% (18.1%–56.7%)	NA	13.3%	100.0%
Dahl 2016	NOVA	Validation	≥4	97.4% (91.1%–99.3%)	23.5% (17.6%–30.5%)	NA	38.0%	95.0%
Lindberg 2022	NOVA	Validation	≥4	100.0% (16.0%–100.0%)	8.3% (3.6%–18.1%)	NA	3.5%	100.0%
Varisco 2026	NOVA	Validation	≥4	87.2% (80.0%–92.5%)	51.4% (46.5%–56.3%)	0.693 (0.655–0.731)	34.9%	93.1%
Zimmermann 2026	NOVA	Validation	≥4	99.1% (96.0%–100.0%)	36.9% (32.0%–42.0%)	NA	29.0%	99.0%

Score thresholds: NOVA ≥4, DENOVA ≥3, MEFIER ≥32 (high-risk stratum). 95% CIs for sensitivity and specificity were calculated using the Wilson score method where not reported in the original publication. AUC values are as originally published; NA = not available (insufficient IE cases for ROC analysis). Studies labelled “derivation” contributed to original score development; all others are independent external validations. For Berge 2019, scores were evaluated separately in the two internal cohorts: the derivation cohort (Skåne University Hospital, Malmö) and the validation cohort (Karolinska University Hospital, Stockholm). EF-BSI = *Enterococcus faecalis* bloodstream infection; IE = infective endocarditis; AUC = area under the receiver-operating characteristic curve; PPV = positive predictive value; NPV = negative predictive value.

The same analysis after exclusion of high RoB studies did not substantially alter the findings (Supplementary Table S6).

### IE predictors

Factors associated with a higher risk of IE in EF-BSI were a higher number of positive blood cultures, community-acquired infection, symptom duration of at least seven days, embolic events, a heart murmur on auscultation, immunosuppression, monomicrobial bacteraemia, prior EF-BSI, prior endocarditis, a prosthetic heart valve, and an unknown portal of entry. Nosocomial acquisition and a urinary source were associated with a lower risk (Supplementary Figure S9).

### Certainty of evidence

Table 4 presents the GRADE assessment. The crude pooled prevalence likely underestimates the true IE burden because of inconsistent echocardiographic coverage; the systematic-imaging subgroup estimate is probably more informative, supported by low risk of bias, absence of heterogeneity, and prospective design. Certainty of evidence was formally moderate, given only two contributing studies and inability to assess publication bias. Although outside the formal GRADE framework, the systematic-imaging estimate is further supported by the meta-regression, which yielded a comparable plateau prevalence while explaining most of the observed between-study variance.

**Table 4:** GRADE summary of findings for IE prevalence and prediction scores’ diagnostic accuracy in EF-BSI.

Outcome	Study design	N. studies (k)	N. participants	Pooled estimate (95% CI)	I^2^	RoB	Inconsistency	Indirectness	Imprecision	Publication bias	Certainty (GRADE)
IE prevalence in EF-BSI (all studies)	Prospective/retrospective cohort studies	15	11,157	11.8% (8.1%–16.8%)	97.3%	Serious	Serious	Not serious	Not serious	Undetected	⊕⊕○○ [LOW]
IE prevalence — systematic echo	Prospective cohort studies	2	887	24.2% (21.5%–27.2%)	0.0%	Not serious	Not serious	Not serious	Serious	Not assessed (k too small)	⊕⊕⊕○ [MODERATE]
IE prevalence — selective echo	Prospective/retrospective cohort studies	12	5570	9.9% (6.4%–14.8%)	95.1%	Serious	Serious	Not serious	Not serious	Undetected	⊕⊕○○ [LOW]
NOVA score — sensitivity	Diagnostic accuracy studies	6	2284	95.6% (88.3%–98.4%)	56.1%	Serious	Serious	Not serious	Not serious	Not assessed (k too small)	⊕⊕○○ [LOW]
NOVA score — specificity	Diagnostic accuracy studies	6	2284	30.7% (21.3%–42.1%)	94.9%	Serious	Serious	Not serious	Not serious	Not assessed (k too small)	⊕⊕○○ [LOW]
DENOVA score — sensitivity	Diagnostic accuracy studies	6	1784	88.7% (78.9%–94.3%)	50.6%	Serious	Serious	Not serious	Not serious	Not assessed (k too small)	⊕⊕○○ [LOW]
DENOVA score — specificity	Diagnostic accuracy studies	6	1784	84.0% (77.8%–88.7%)	84.4%	Serious	Serious	Not serious	Not serious	Not assessed (k too small)	⊕⊕○○ [LOW]
MEFIER score — sensitivity	Diagnostic accuracy studies	2	3084	54.5% (24.9%–81.3%)	84.6%	Serious	Serious	Not serious	Serious	Not assessed (k too small)	⊕⊕○○ [LOW]
MEFIER score — specificity	Diagnostic accuracy studies	2	3084	81.0% (66.2%–90.2%)	95.1%	Serious	Serious	Not serious	Serious	Not assessed (k too small)	⊕⊕○○ [LOW]

Certainty ratings: ⊕⊕⊕⊕ High; ⊕⊕⊕○ Moderate; ⊕⊕○○ Low; ⊕○○○ Very low. EF-BSI = *Enterococcus faecalis* bloodstream infection; GRADE = Grading of Recommendations Assessment, Development and Evaluation; IE = infective endocarditis; CI = confidence interval; k = number of studies; RoB = risk of bias.

### Publication bias

Visual inspection of the funnel plot suggested a low probability of publication bias (Supplementary Figure S10); Egger's test showed no asymmetry (p = 0.36), and the trim-and-fill method identified no missing studies.

## Discussion

To our knowledge, this is the first systematic review and meta-analysis to estimate the prevalence of IE in EF-BSI and to model the dependence of that estimate on echocardiographic ascertainment. Our main finding is that the widely discordant prevalence estimates reported in the literature are not contradictory but largely reconcilable: the lower figures from routine practice largely reflect incomplete echocardiographic ascertainment rather than a genuinely lower underlying disease burden.

Near-total heterogeneity (I^2^ 97.3%) renders the crude pooled estimate (11.8%, 95% CI 8.1–16.8) clinically uninterpretable in isolation. That heterogeneity was, however, explicable rather than random: echocardiographic coverage accounted for most of the between-study variance, and once ascertainment was complete the estimates converged. The two cohorts that imaged every patient gave closely concordant results (24.2%, 95% CI 21.5–27.2; I^2^ = 0.0%),^2^^,^^14^ and the meta-regression projected a plateau of comparable magnitude (≈30%) as coverage approached completeness. These two independent lines of evidence therefore converge on an ascertainment-adjusted prevalence of 24–30%, comparable to or higher than that of *Staphylococcus aureus* bacteraemia (SAB).^23^ The markedly lower estimates that dominate the retrospective literature are largely artefacts of incomplete imaging. The gradient spanned the full dataset, from the lowest prevalences in cohorts with the sparsest imaging or the most selected populations (1–4%)^11^^,^^15^ to 23–26% in the highest-coverage cohorts irrespective of design.^2^^,^^14^^,^^19^ The ascertainment-coverage relationship was dose-dependent and robust to model specification (R^2^ 57.8–76.6% linear; up to 91.5% quadratic); the quadratic is best read as describing a prevalence that rises and then flattens within the observed coverage range, rather than a precisely located asymptote. The model-projected plateau (≈30%) approximates the directly estimated systematic-imaging subgroup (24.2%), which requires no extrapolation. The relationship held under both the Duke–ISCVID 2023 and the modified Duke 2000 definitions, indicating a structural feature of the literature rather than an effect of the more inclusive criteria. Conditioning on coverage reduced between-study variance by approximately 90% (τ^2^ 0.5949 to ≈0.05); residual I^2^ remained high because the cohorts are large enough for even small residual variance to be statistically detectable, not because substantial unexplained clinical heterogeneity remained. The EfFAECT cohort further isolates the criterion effect: applying Duke–ISCVID rather than modified Duke criteria reclassified definite IE from 13.3% to 23.0% in the same patients with the same imaging.^14^ The criterion effect is thus separable from ascertainment. Our adjusted estimate is concordant with the prospective benchmarks (26% and 23%),^2^^,^^14^ suggesting that low retrospective figures reflect how intensively IE was sought rather than how often it occurred.

Among the included studies, only Dahl et al. compared outcomes by echocardiographic status: no death or bacteraemia relapse occurred among the few unimaged patients in their otherwise near-universal-echo cohort, and this null finding was used to justify a lower adjusted prevalence (24.1% vs 26.1%).^2^ No comparable analysis is available for the other included studies, and this residual is in any case small and favourably selected, unlikely to generalise to the much larger and differently selected unimaged fractions (60–80%) in the selective-echo cohorts. The clinical implication is direct. In the testing-threshold framework of clinical decision theory,^24^ echocardiography is justified once the probability of disease exceeds a definable threshold. In the analogous SAB decision analysis, this threshold is approximately 1.1%: above this probability of occult IE, an echocardiography-guided strategy yields the best survival.^25^ No testing threshold has been formally derived for EF-BSI, but a prevalence of 24–30% lies more than an order of magnitude above the SAB-derived benchmark. Current ESC guidelines recommend that echocardiography be considered in EF-BSI (Class IIa)^8^; our estimate, however, suggests this could reasonably move from a selective to a routine recommendation. The data do not, however, indicate which modality should be applied to whom. TEE is the more sensitive and definitive test — TTE missed approximately half of vegetations in the prospective enterococcal cohort,^2^ so routine imaging should not be equated with TTE alone. Routine TEE in every patient is nonetheless neither supported by our data nor feasible in most settings. A pragmatic approach, to be tailored to local resources and validated prospectively, would be TTE in all patients with TEE reserved as a second-line study for those at higher risk, namely patients with a positive prediction score, a negative or inconclusive TTE with persistent clinical suspicion, or specific high-risk features such as prosthetic valve or prior endocarditis.

The clinical predictors of IE were consistent and biologically coherent. Most, however, were informed by only one or two cohorts, and the pooled estimates combined adjusted and unadjusted associations and, where only hazard ratios were available, treated these as approximate odds ratios. Among the seven pooled factors, the most robust were a prosthetic valve (OR 4.78, 95% CI 3.08–7.42) and community acquisition (OR 3.11, 95% CI 1.96–4.94), both without heterogeneity (I^2^ = 0.0%). Previous IE (OR 5.86) and an unknown portal of entry (OR 3.59) were positively associated but imprecise (I^2^ 58.4–73.9%), whereas a cardiac device, any pre-existing valve disease, and male sex were not significant. Among single-cohort factors, a heart murmur on auscultation, a symptom duration of at least seven days, embolic phenomena, a higher number of positive blood cultures, immunosuppression, monomicrobial bacteraemia, and previous EF-BSI were each positively associated, while age and fever were not. Nosocomial or healthcare-associated acquisition and a urinary source were inversely associated (Supplementary Figure S9). These estimates corroborate the independent predictors of the largest prospective EF-BSI cohort.^2^ They are broadly consistent with the risk architecture of SAB, where a parallel meta-analysis identified prosthetic valves, previous IE, embolic events, and intracardiac devices as the strongest associations.^26^ Several predictors overlap the components of NOVA and DENOVA, defining the pre-test probability of IE in both directions.^5^^,^^10^ However, expressed as odds ratios rather than likelihood ratios, they cannot be translated into individual post-test probabilities or used to identify a subgroup at sufficiently low risk to forgo imaging. Whether a genuinely low-risk subgroup can be defined for EF-BSI, as proposed on retrospective evidence for SAB,^27^^,^^28^ remains to be established in prospective, outcome-based studies.

At a pre-test probability of 24–30%, the question is no longer whether to image but how to sequence and target imaging; our pooled accuracy data suggest that existing scores do not define a subset safe to defer. DENOVA (≥3)^10^ achieved pooled sensitivity of 88.7% (78.9–94.3; I^2^ = 50.6%) and specificity of 84.0% (77.8–88.7; I^2^ = 84.4%). NOVA (≥4)^5^ combined high sensitivity with low specificity and so classified few patients as low risk, and MEFIER^11^ demonstrated low sensitivity, limiting its use as a screening tool. However, this estimate relies on only two studies. NOVA and DENOVA represent complementary trade-offs: NOVA's near-maximal sensitivity leaves a very small actionable low-risk stratum at its standard cutoff,^5^ whereas DENOVA's higher specificity comes at the cost of a validation sensitivity substantially below derivation.^10^^,^^14^^,^^22^ Few studies per score precluded summary-ROC modelling, and pooled sensitivity is weighted by derivation cohorts reporting near-100% values, so these estimates are optimistic and provisional. This optimism does not survive prospective validation. In the largest prospective study, an international cohort of 543 patients undergoing protocol echocardiography, DENOVA ≥3 had a sensitivity of 79.2% and a negative predictive value (NPV) of 93.0%, missing 26 of 125 IE cases.^14^ The fall from derivation reflects in-sample optimism, incorporation bias, modest inter-rater reliability,^29^ and most specifically the greater sensitivity of the Duke–ISCVID 2023 criteria,^30^ against which the score was not derived. This figure also overstates the score's triage value: in the Varisco et al. cohort, 84 patients had a diagnostically positive TTE (83 with IE), so sensitivity was partly estimated in patients already diagnosed.^14^^,^^31^ In practice a score would guide TEE only among patients with a negative or inconclusive TTE, in whom its performance is unestablished and probably lower,^31^ and its decision-curve net benefit attenuates at lower prevalence.^32^ The key clinical question is whether a negative score can reliably exclude IE at the 24–30% prevalence we have derived — not merely identify those less likely to have it. At the corresponding negative likelihood ratio of approximately 0.25, a negative DENOVA still leaves a 7–10% probability of IE at a 24–30% pre-test prevalence — far exceeding the SAB-derived threshold.^25^ A low score therefore lowers but does not abolish the indication for imaging. Prediction scores should prioritise and sequence TEE rather than authorise its omission, and any score-guided deferral strategy requires prospective, outcome-based validation. A useful rule would need a higher NPV, calibrated to the Duke–ISCVID 2023 definition; derivation candidates might incorporate predictors absent from NOVA and DENOVA, such as prosthetic valve, prior IE, intracardiac devices, and blood-culture time-to-positivity.^11^^,^^19^ DENOVi illustrates this route, adding intracardiac devices and removing the subjective murmur item, with favourable rule-out metrics in retrospective data but no prospective validation.^33^ Either route must guard against overfitting and incorporation bias, and any candidate requires prospective derivation and external validation against protocolised echocardiography.

Several limitations apply. The systematic-imaging subgroup comprised only two cohorts, so the direct estimate, although internally consistent, may be imprecise, and the meta-regression plateau is anchored by few high-coverage studies. Echocardiographic coverage does not capture imaging quality or operator expertise, and its correlation with prospective design and contemporary diagnostic criteria means the meta-regression cannot fully isolate imaging from these covariates — a constraint inherent to study-level inference and the reason we rest the 24–30% range on triangulation rather than on the regression alone. None of the selective-echo studies compared clinical outcomes by echocardiographic status; the only such comparison among the included studies was in a systematic-echo cohort,^2^ discussed above. Because echocardiographic evidence of endocardial involvement is a major Duke criterion, a definite IE diagnosis without any imaging is rarely possible; the frequency of IE among unimaged patients is therefore largely unobserved rather than merely unreported, and we cannot exclude that patients selected for imaging differ in underlying risk from those who are not. The two cohorts with a systematic imaging strategy provide the closest available approximation to this counterfactual. The included cohorts were clinically and methodologically heterogeneous, encompassing registry/ICD-10 ascertainment,^11^^,^^18^ a mid-study change of diagnostic criteria,^4^ and a cohort confined to oncohaematological patients.^15^ Small-study effects could not be reliably excluded: Egger's test was non-significant (p = 0.42) but is underpowered at this number of studies and degree of heterogeneity, and any funnel asymmetry is more plausibly explained by the coverage gradient than by publication bias. The pooled accuracy of prediction scores should be read cautiously: with at most five studies per score, sensitivity and specificity were pooled separately rather than within a bivariate model, so these estimates are correspondingly approximate. Individual patient data were not available, precluding patient-level meta-regression to adjust for simultaneous covariates. Sex-disaggregated data were not consistently reported across included studies and could not be pooled; the generalisability of findings across sexes remains unestablished. Finally, no cost-effectiveness analysis or randomised comparison of imaging strategies exists for EF-BSI. Overall certainty (GRADE) was low to moderate.

Once echocardiographic ascertainment is accounted for, IE complicates approximately one quarter of EF-BSI episodes. The markedly lower figures reported in routine practice are largely artifacts of incomplete imaging, not evidence of lower disease frequency. Together with the reclassification of EF as a typical endocarditis organism under the 2023 Duke–ISCVID criteria, this supports a shift from selective to routine echocardiography in EF-BSI. Because TTE alone is insufficient to exclude endocarditis, TEE remains the definitive test. How best to sequence the two modalities and target the more invasive study, including the role of prediction scores, requires prospective evaluation and will depend on local resources. Existing prediction scores can prioritise and expedite imaging, but on current evidence they cannot safely define a subgroup in whom it may be omitted, and a low score should not override clinical suspicion. The immediate research priorities are the prospective derivation and validation of a more sensitive, Duke–ISCVID-calibrated prediction rule and the identification of a genuinely low-risk subgroup. Pending such evidence, including cost-effectiveness data, our findings argue against omitting echocardiography in EF-BSI.

## Contributors

G.M. initiated and supervised the study. G.M. and N.S. developed the study methodology and design. N.S. and G.M. screened abstracts and titles for inclusion and assessed the studies at full-text level. Al.Ca. resolved any discordance. N.S. extracted the data, and G.M. and An.Co. independently checked and verified the data. G.M. and N.S. conducted the study quality assessment, and Al.Ca. resolved any conflicts. N.S. performed the statistical analyses. G.M. and N.S. drafted the manuscript. V.R., M.L., A.M., R.B., F.O., and G.L. critically revised the manuscript for important intellectual content. All authors reviewed the final version of the manuscript and approved it for submission.

## Data sharing statement

The study protocol was prospectively registered in PROSPERO on 3 April 2026 (registration number CRD420251240242). The Supplementary Materials include the full methods section and additional study data, including supporting materials for the systematic review and meta-analysis. Further information is available from the corresponding author upon reasonable request.

## Declaration of interests

The authors declare no conflicts of interest related to this study.

## Figures and Tables

**Supplementary Figure S1:**
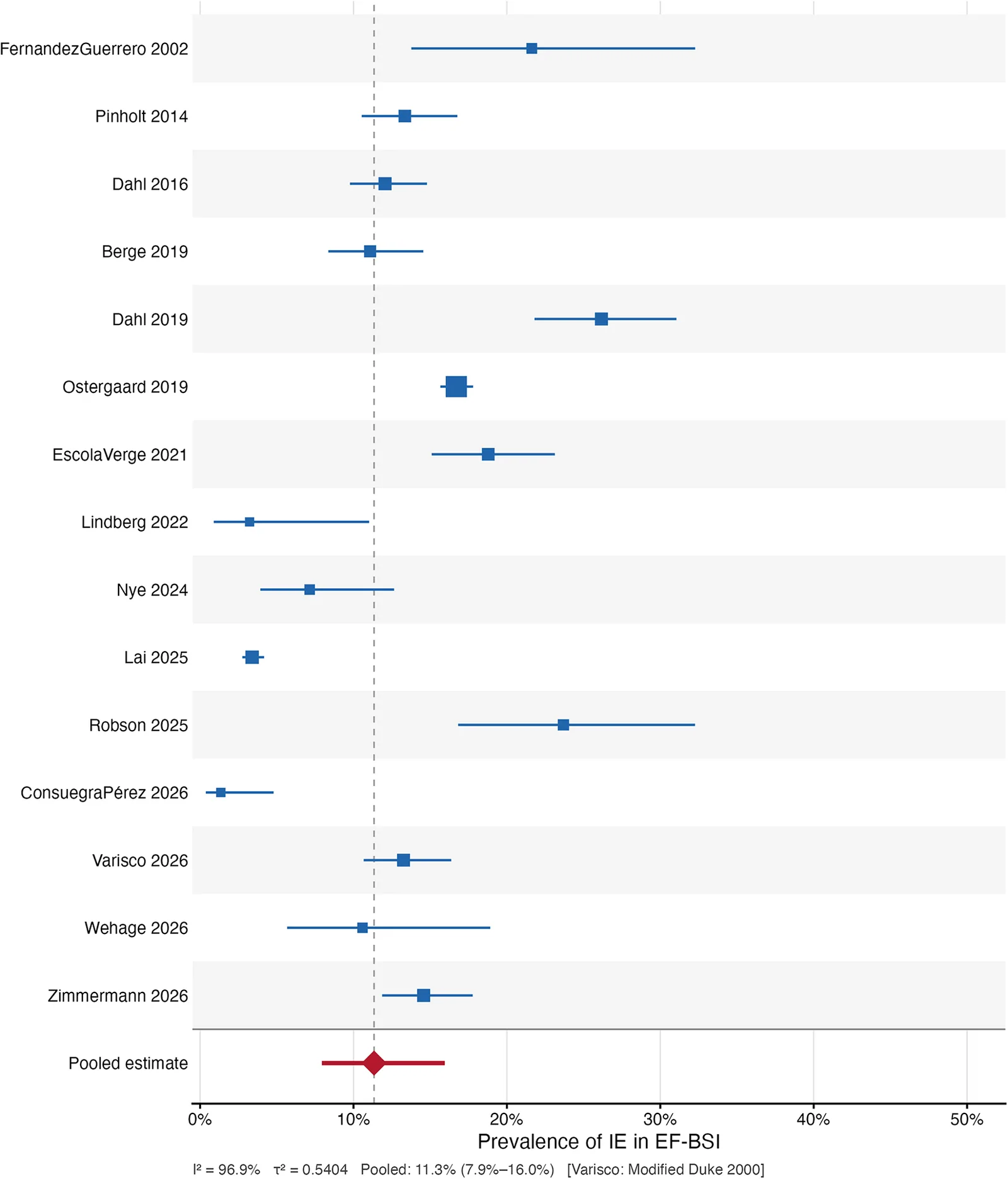


**Supplementary Figure S2:**
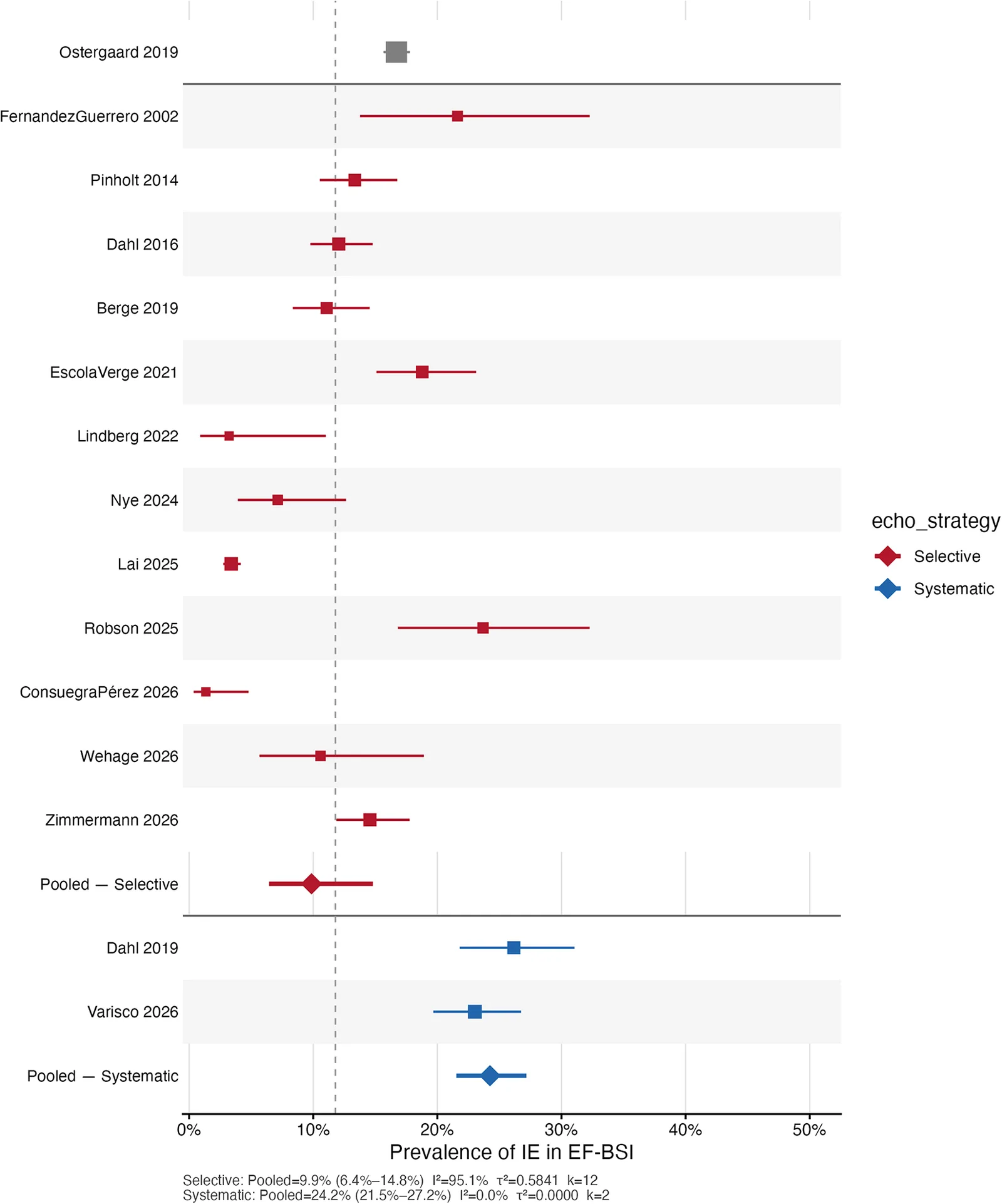


**Supplementary Figure S3:**
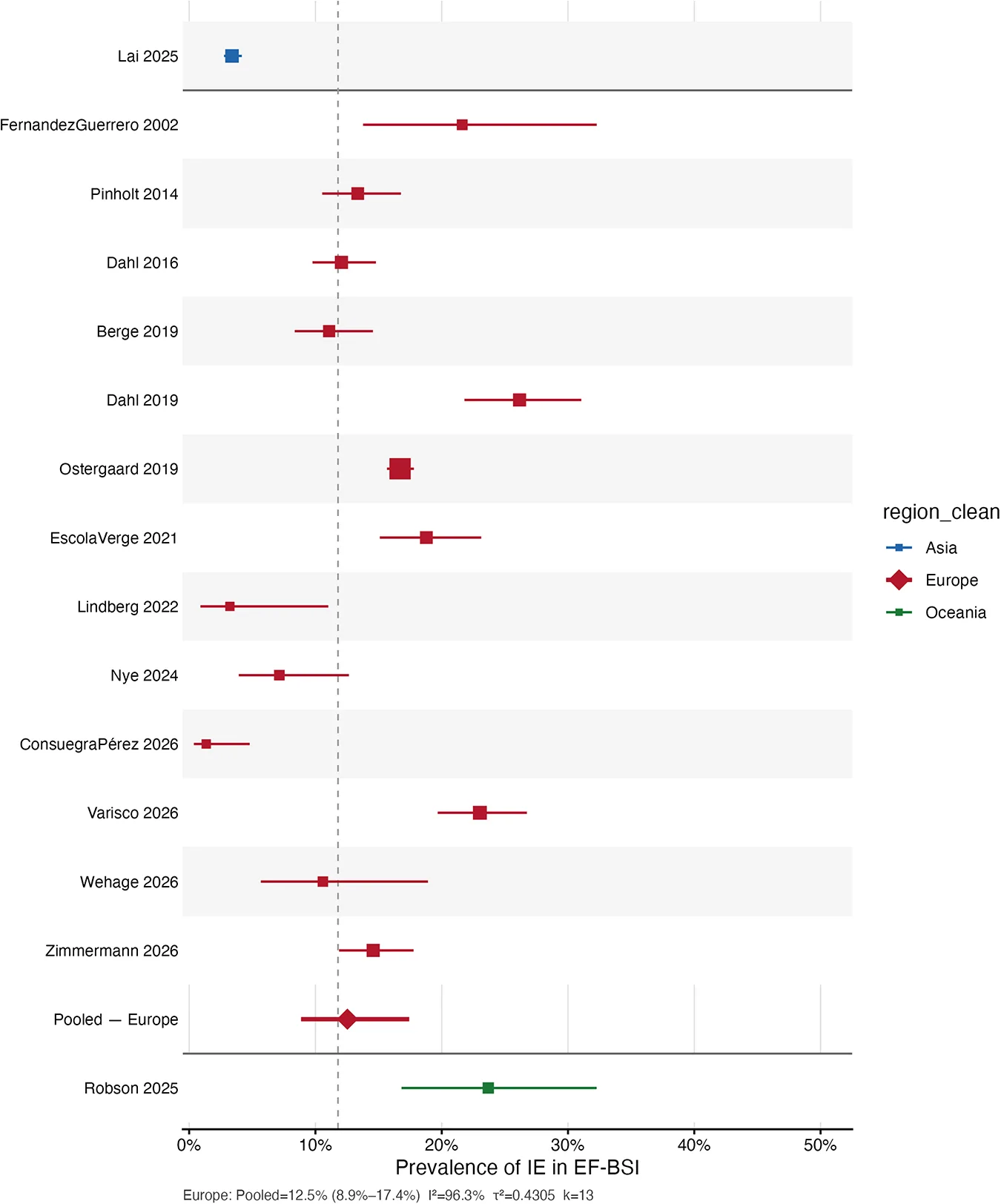


**Supplementary Figure S4:**
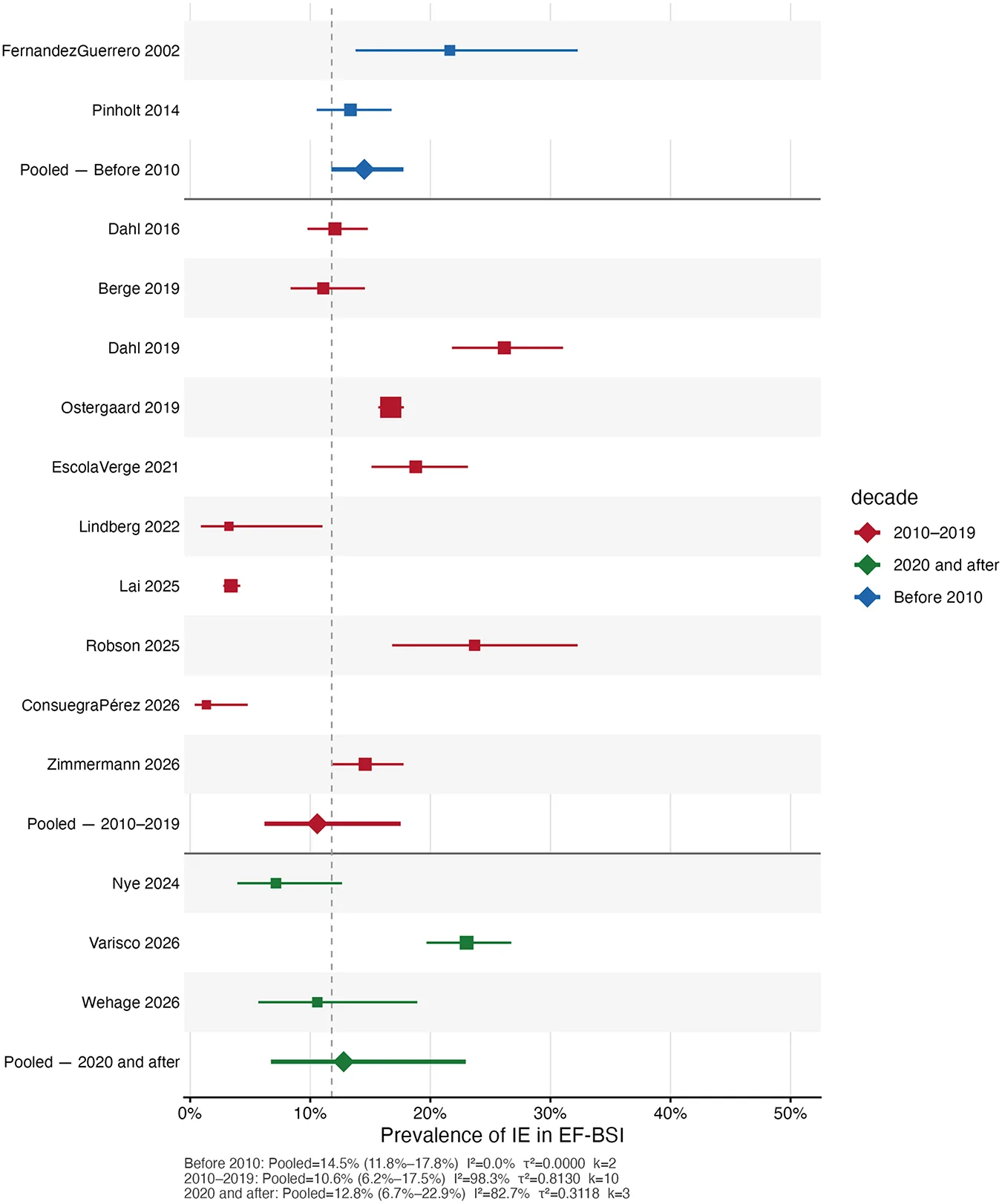


**Supplementary Figure S5:**
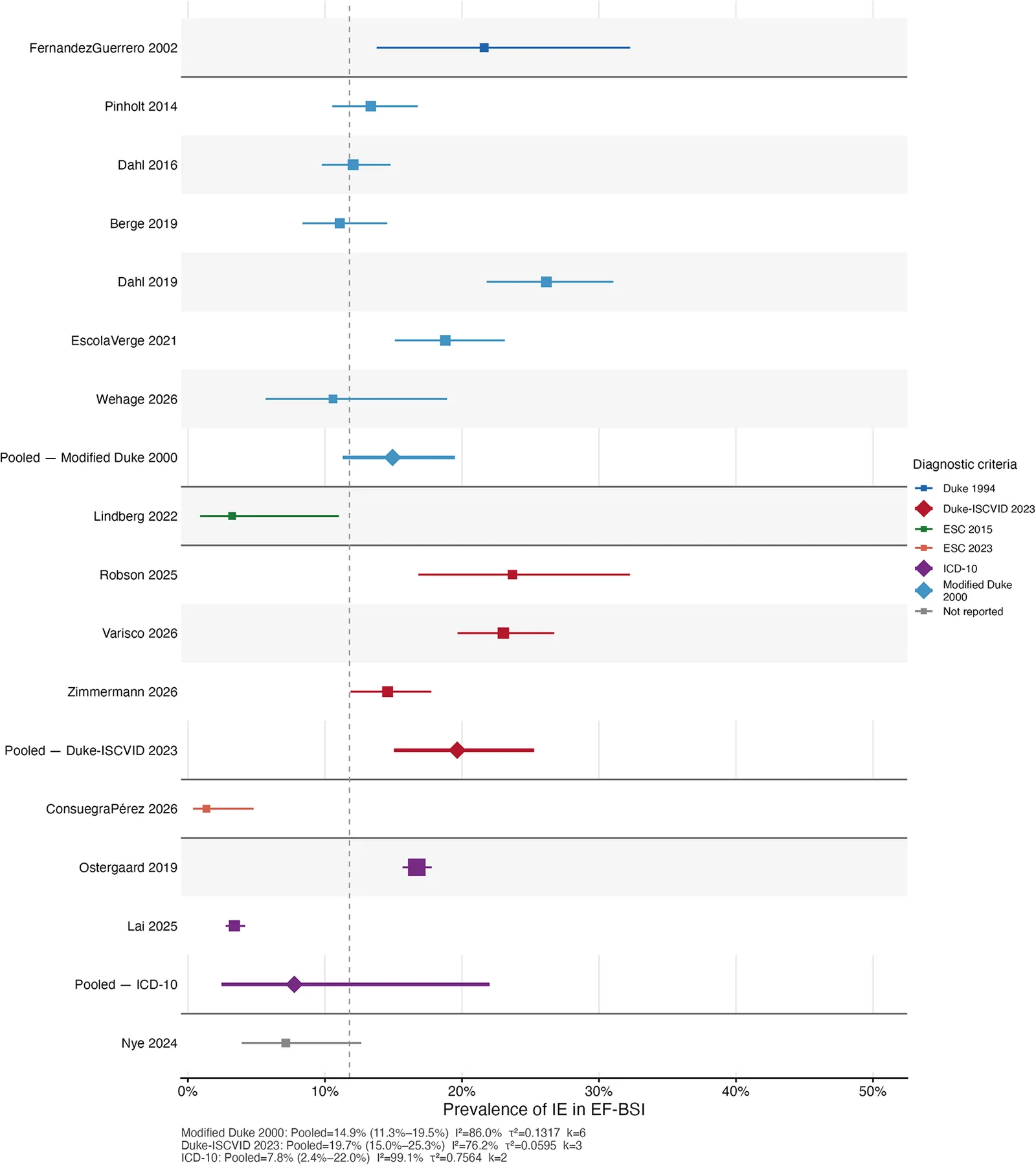


**Supplementary Figure S6:**
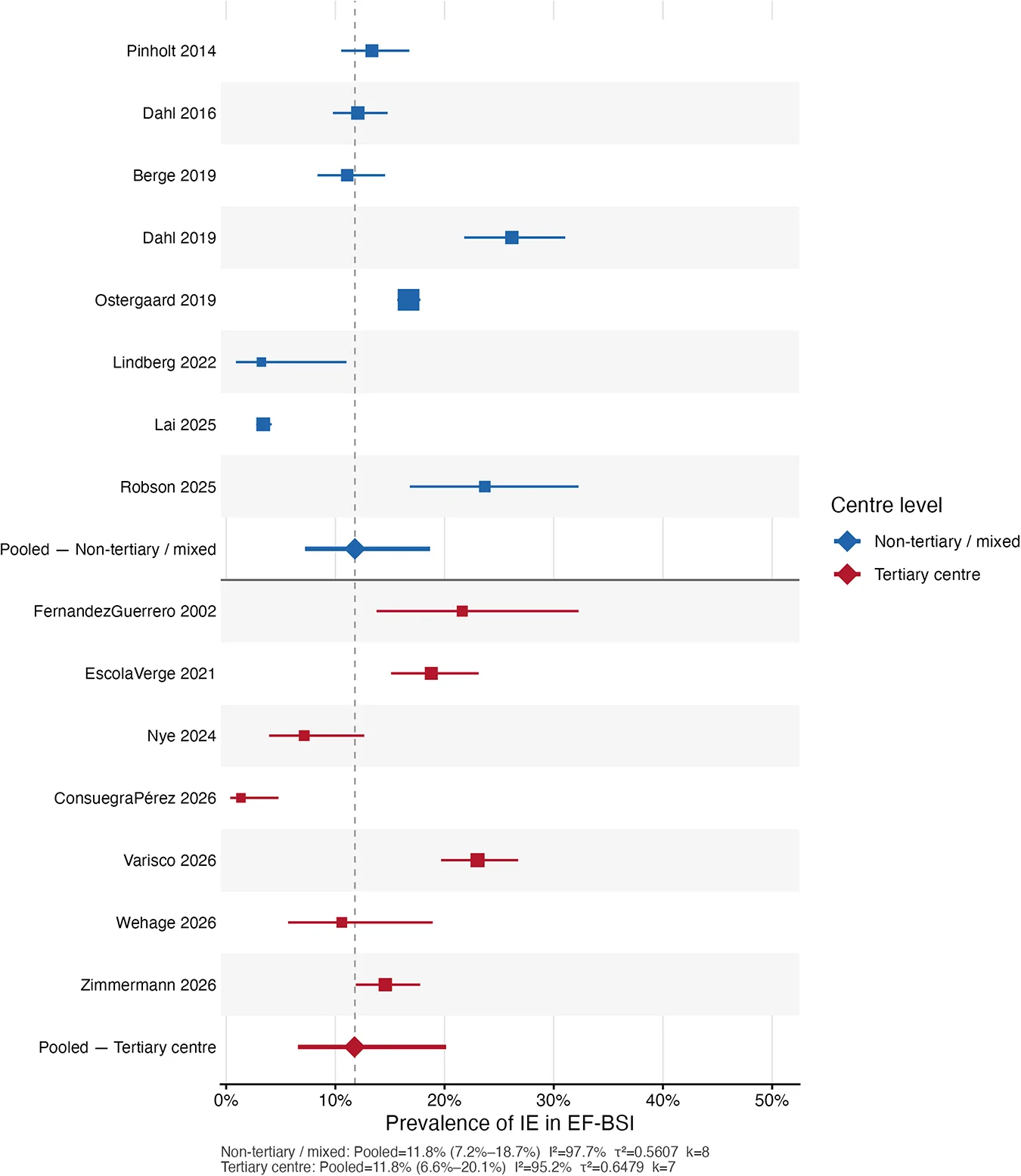


**Supplementary Figure S7:**
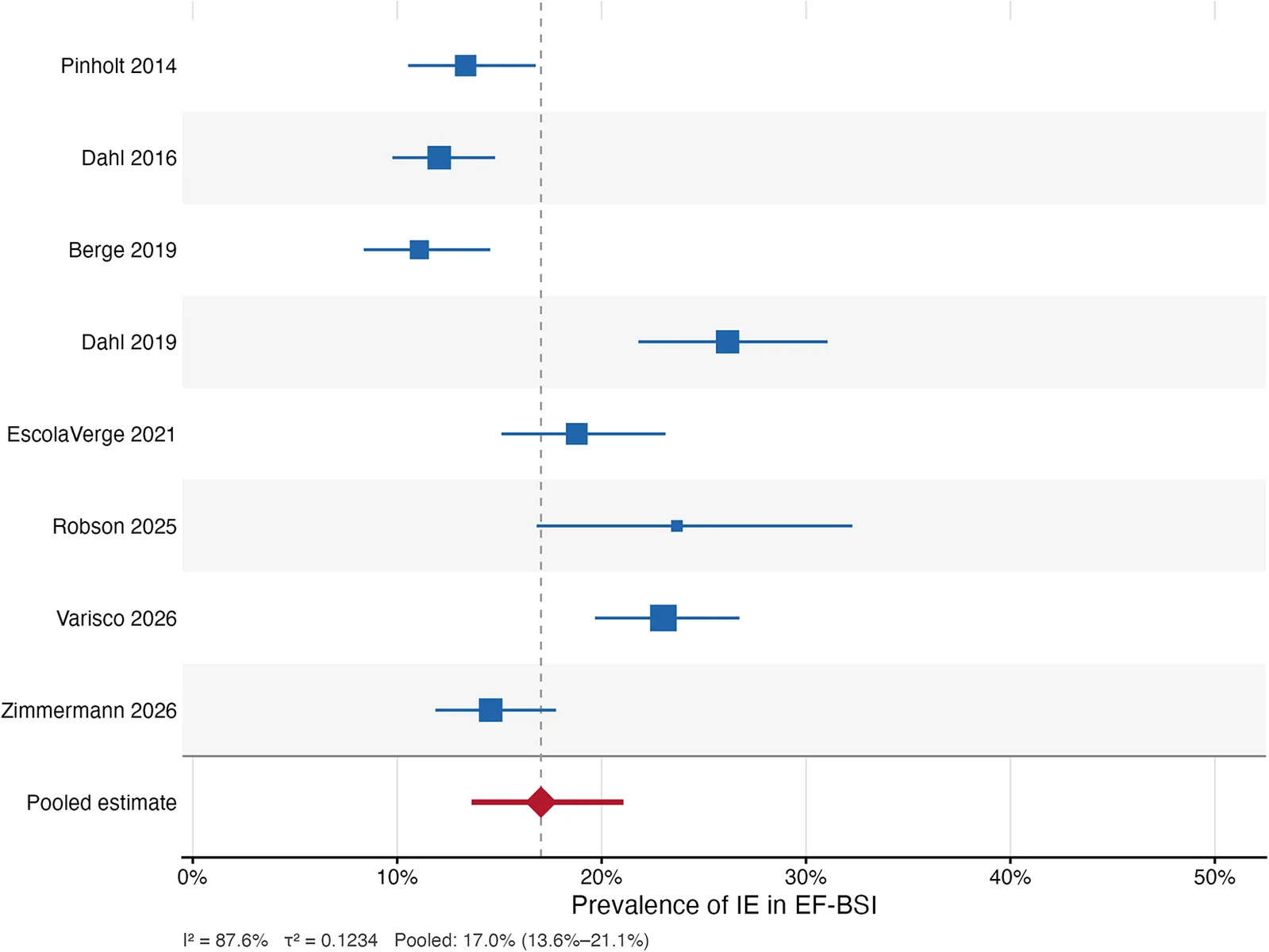


**Supplementary Figure S8:**
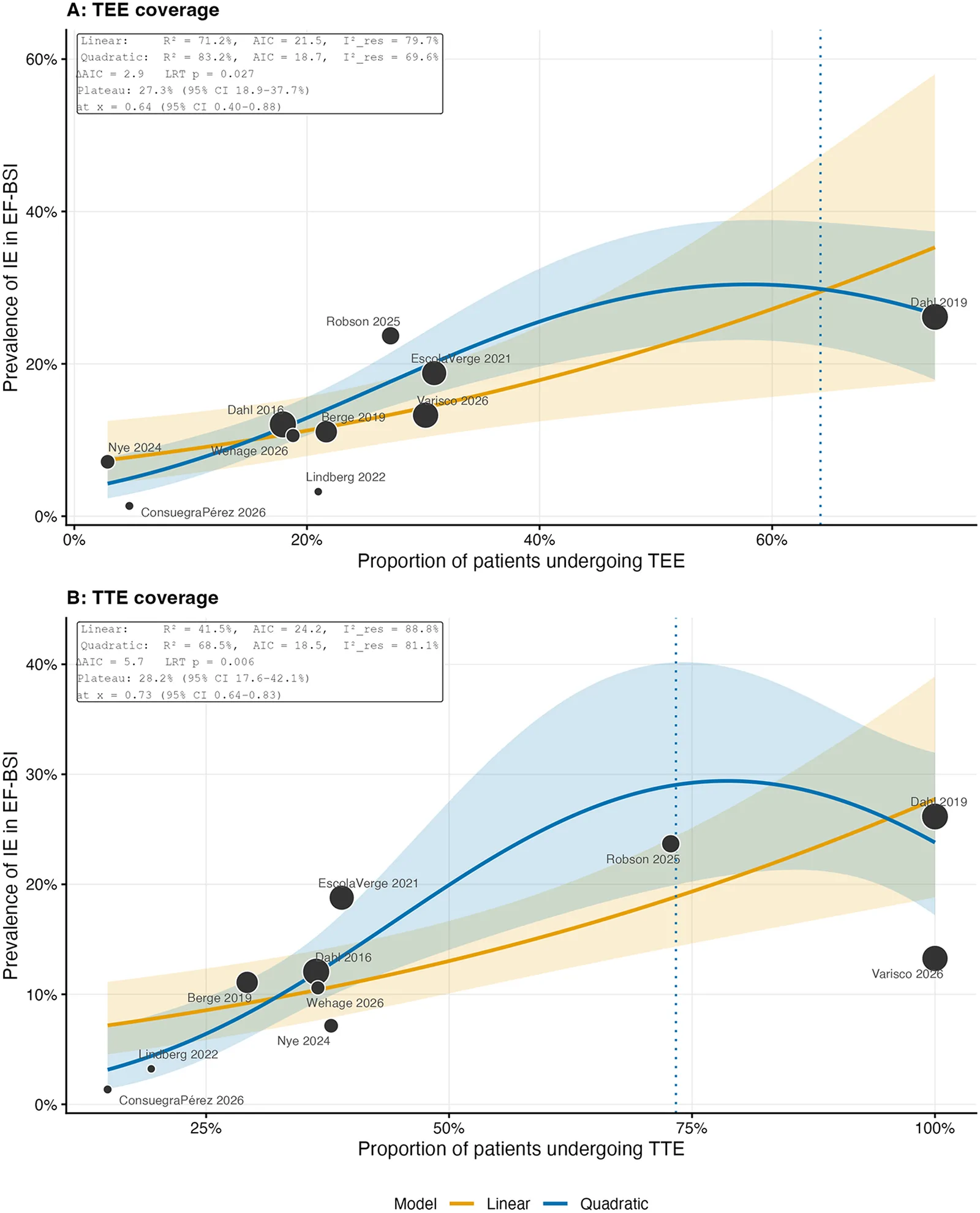


**Supplementary Figure S9:**
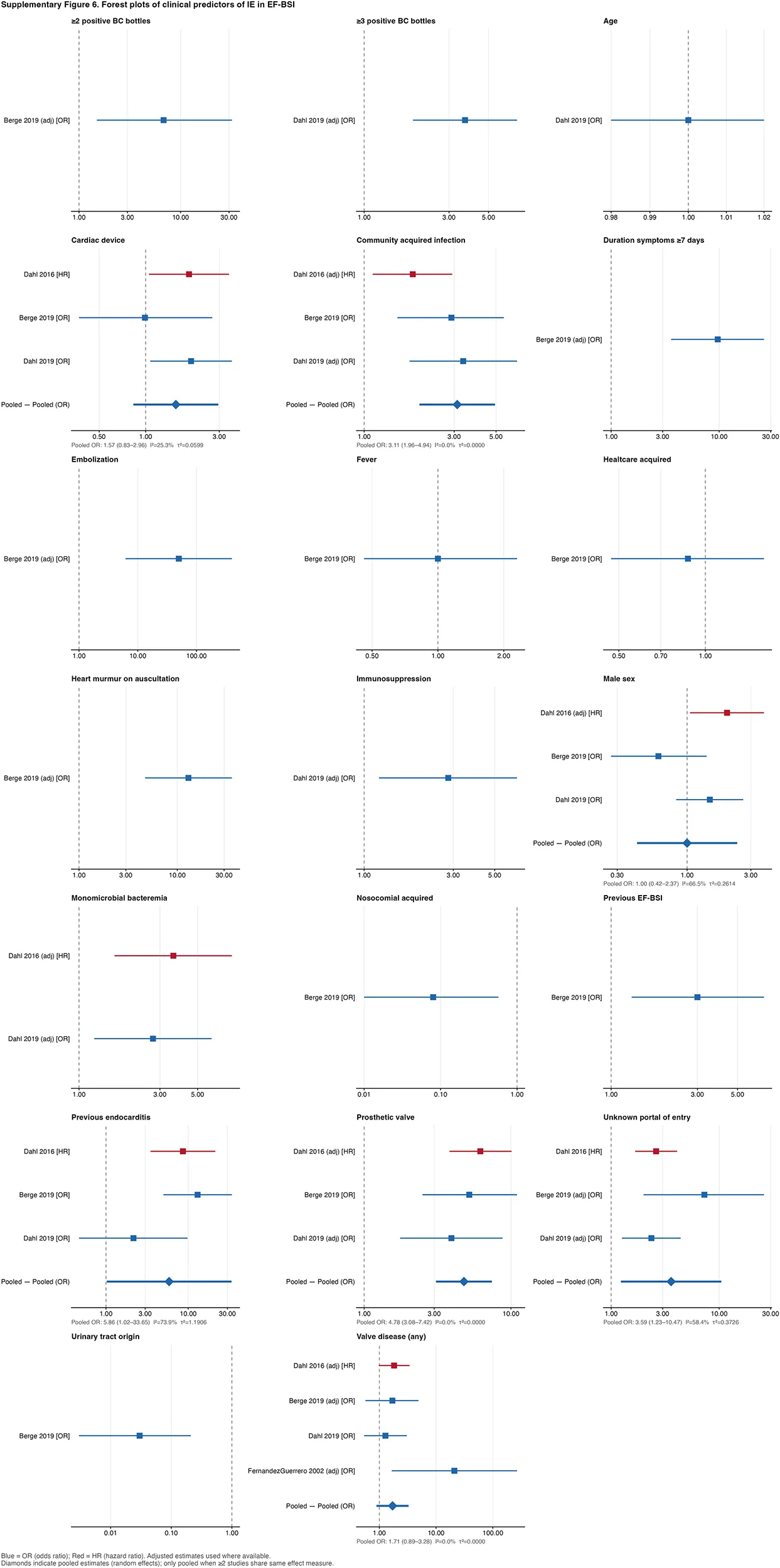


**Supplementary Figure S10:**