# Supplementary material for: Enterococcus faecalis bacteraemia and infective endocarditis: a systematic review and meta-analysis
Source: eClinicalMedicine. 2026 Sep 22;101:104241. doi: 10.1016/j.eclinm.2026.104241 (PMC13688461; doi:10.1016/j.eclinm.2026.104241)
Supplement: Supplementary file 1 [file mmc1.docx]

**Supplementary Material**

*Infective Endocarditis in Enterococcus faecalis Bloodstream Infection: A Systematic Review and Meta-Analysis of Prevalence, Risk Factors, and Prediction Scores*

Giovanni Mori, Alessandro Castelli, Andrea Cordin, Giovanni Lorenzin, Roberto Bonmassari, Francesco Onorati, Alberto Maino, Massimiliano Lanzafame, Vito Racanelli, Nicola Susca

**Table of contents**

**Supplementary Methods**

**Supplementary Figure 1.** Forest plot — sensitivity analysis (Modified Duke 2000 criteria)

**Supplementary Figure 2.** Forest plot — echocardiographic strategy

**Supplementary Figure 3.** Forest plot — geographical region

**Supplementary Figure 4.** Forest plot — study enrolment period

**Supplementary Figure 5.** Forest plot — IE diagnostic criteria

**Supplementary Figure 6.** Forest plot — centre level

**Supplementary Figure 7.** Forest plot — sensitivity analysis (high Risk of Bias excluded)

**Supplementary Figure 8.** Meta-regression — sensitivity analysis (Modified Duke 2000 criteria)

**Supplementary Figure 9.** Forest plots — clinical predictors of IE

**Supplementary Figure 10.** Funnel plot — publication bias

**Supplementary Table 1.** Risk of bias assessment (QUIPS) of the included studies

**Supplementary Table 2.** Risk-of-bias sensitivity analysis — IE prevalence

**Supplementary Table 3.** *Meta-regression coefficients — sensitivity analysis (Varisco: Modified Duke 2000)*

**Supplementary Table 4.** Leave-one-out sensitivity analysis

**Supplementary Table 5.** Influence analysis

**Supplementary Table 6.** Risk-of-bias sensitivity analysis — prediction scores

**Studies excluded after full-text assessment**

**PRISMA 2020 Checklist**

**SUPPLEMENTARY METHODS**

**Eligibility criteria**

Observational studies (retrospective or prospective) reporting the prevalence of IE in patients with microbiologically confirmed EF-BSI were eligible, irrespective of the study's primary objective. The primary outcome was the prevalence of IE among EF-BSI episodes, regardless of the diagnostic criteria used. The following were excluded: studies of mixed bacterial populations in which EF-BSI episodes or EF-IE could not be separated, conference abstracts, case-control designs, IE-enriched denominators, secondary reanalyses, and overlapping cohorts. Studies without systematic echocardiography or using non-standard diagnostic criteria (e.g., ICD-10 coding) were retained and addressed through stratified or sensitivity analyses.

**Literature search**

PubMed, Scopus, Web of Science, and Embase were searched from inception to 20 January 2026 without language restriction, and the search was subsequently re-run on 16 August 2026 using the identical strategy to capture records added after the primary search, with the exception of Embase. The PubMed search string was: (("Enterococcus faecalis"[Mesh] OR "Enterococcus faecalis"[tiab] OR "E. faecalis"[tiab]) AND ("Bacteremia"[Mesh] OR "bacteremia"[tiab] OR "bacteraemia"[tiab] OR "bloodstream infection"[tiab] OR "bloodstream infections"[tiab] OR "BSI"[tiab] OR "blood culture"[tiab]) AND ("Endocarditis"[Mesh] OR "Endocarditis, Bacterial"[Mesh] OR "endocarditis"[tiab] OR "infective endocarditis"[tiab])) OR (("NOVA score"[tiab] OR "DENOVA score"[tiab] OR "DENOVA"[tiab] OR "MEFIER"[tiab]) AND ("Enterococcus"[tiab] OR "enterococcal"[tiab])). Equivalent strings were adapted for the other databases. Reference lists of full-text articles were screened for additional citations, and further articles were identified from the authors' personal files. Rayyan (rayyan.ai) was used to facilitate title and abstract screening. Two authors (G.M. and N.S.) independently screened titles and abstracts and selected articles for full-text review; disagreements were resolved by a third author (Al.Ca.).

**Data extraction**

Data were extracted into a spreadsheet across the following domains: study characteristics; population and outcomes; predictors of IE and their effect estimates; IE prediction scores and their diagnostic performance; risk of bias; and subgroup data for the stratified analyses. Where not reported in the original publication, 95% confidence intervals were calculated using the Wilson score method. In the Varisco et al. study, which reported IE under both frameworks, cases defined by the Duke–ISCVID 2023 criteria were used in the main analyses and those defined by the modified Duke 2000 criteria in the sensitivity analyses. N.S. extracted the data, and G.M. and An.Co. independently checked and verified the data.

**Risk of bias assessment**

Risk of bias (RoB) was assessed with the Quality in Prognostic Studies (QUIPS) tool. The tool comprises six domains: study participation, attrition, prognostic factor measurement, outcome measurement, confounding, and statistical analysis. Each domain, and the study overall, was rated as low, moderate, or high risk of bias. N.S. and G.M. conducted the study quality assessment, and Al.Ca. resolved any conflicts.

**Statistical analysis**

The pooled IE prevalence was estimated with a generalized linear mixed model (GLMM) with a logit link, which models the within-study binomial distribution of IE cases and accounts for between-study heterogeneity through a study-level random effect (Laplace approximation). Heterogeneity was quantified by I² and the between-study variance by τ², both estimated by maximum likelihood within the GLMM. The pooled estimate was back-transformed from the logit scale for presentation.

A meta-regression was performed with TEE and TTE coverage as separate moderators using the rma function in the metafor package, with logit-transformed prevalences and within-study variances estimated using the delta method. Linear and quadratic models were assessed for each moderator and compared by explained variance (R²), Akaike Information Criterion (AIC), and the likelihood ratio test (LRT). The REML estimator was used for coefficient and R² estimation; for AIC comparison and LRT, models were refitted with the ML estimator, as REML-based models are not directly comparable on these measures. A negative quadratic coefficient yields a concave-down curve whose vertex corresponds to the maximum fitted IE prevalence; this was interpreted as the model-estimated plateau prevalence under near-complete echocardiographic coverage, together with the corresponding coverage level. Both values were back-transformed from the logit scale for presentation.

A leave-one-out (LOO) sensitivity analysis refitted each model after omitting one study at a time to assess the stability of the estimates and their dependence on individual studies. All main analyses and meta-regressions were repeated substituting the modified Duke 2000 criteria for the Duke–ISCVID 2023 criteria in the Varisco et al study.

Diagnostic performance of NOVA, DENOVA, and MEFIER was summarised as sensitivity, specificity, and area under the ROC curve (AUROC). For NOVA and DENOVA, pooled sensitivity and specificity were estimated as proportions on the logit scale using random-effects models (REML), using the number of true positives over the total number of IE-positive patients and the number of true negatives over the total number of IE-negative patients as inputs, respectively. AUROC values were pooled after logit transformation with within-study variance approximated from reported 95% confidence intervals.

The main analysis was stratified by echocardiographic strategy (systematic vs selective), geographic region, study period (based on the median recruitment year), diagnostic criteria, centre level (tertiary centre vs non-tertiary/mixed), and RoB. Predictors of IE were summarised as odds ratios (OR) or hazard ratios (HR), using adjusted estimates where available; when the same predictor was reported in more than one study, a pooled estimate was obtained with a random-effects model (REML), with effect sizes displayed on a logarithmic scale.

**Publication bias**

Publication bias was assessed by visual inspection of a funnel plot of IE prevalence (logit scale) vs standard error, Egger's test, and the trim-and-fill method.

**Evidence strength**

The certainty of evidence was rated with the GRADE framework (Grading of Recommendations Assessment, Development and Evaluation) for IE prevalence, echocardiographic strategy, and the sensitivity and specificity of the prediction scores, across the domains of risk of bias, inconsistency, imprecision, indirectness, and publication bias. A two-sided p<0·05 was considered statistically significant. Analyses were performed in R (version 4.2.3) using the metafor, ggplot2, patchwork, dplyr, tidyr, scales, ggrepel, officer, and DiagrammeR packages.

**FIGURE LEGENDS**

**Supplementary Figure 1.** *Forest plot of the pooled prevalence of IE in EF-BSI — sensitivity analysis substituting the modified Duke 2000 criteria for the Duke–ISCVID 2023 criteria in the Varisco et al (2026) study*.

All other studies and conventions as in Figure 2. I² = 96·9%; τ² = 0·5404; pooled prevalence 11·3% (95% CI 7·9–16·0%).

IE = infective endocarditis; EF-BSI = *Enterococcus faecalis* bloodstream infection; I² = between-study heterogeneity statistic; τ² = estimated between-study variance.

**Supplementary Figure 2.** *Forest plot of IE prevalence in EF-BSI stratified by echocardiographic strateg*y.

Studies using a systematic strategy (TTE in all patients) are shown in blue; studies using a selective strategy are shown in red. Ostergaard et al (2019) (grey, top) used a registry-based ascertainment approach and did not report a formal echocardiographic strategy; it was excluded from both subgroup pooled estimates. Selective: pooled 9·9% (95% CI 6·4–14·8%), I²=95·1%, τ²=0·5841, k=12. Systematic: pooled 24·2% (95% CI 21·5–27·2%), I²=0·0%, τ²=0·0000, k=2. Diamonds represent subgroup pooled estimates.

IE = infective endocarditis; EF-BSI = *Enterococcus faecalis* bloodstream infection; TTE = transthoracic echocardiography.

**Supplementary Figure 3.** *Forest plot of IE prevalence in EF-BSI stratified by geographical region.*

Europe (red): pooled 12·5% (95% CI 8·9–17·4%), I²=96·3%, τ²=0·4305, k=13. Asia (blue, Lai 2025) and Oceania (green, Robson 2025) each contributed a single study; no subgroup pooled estimate was generated for these regions.

IE = infective endocarditis; EF-BSI = *Enterococcus faecalis* bloodstream infection

**Supplementary Figure 4.** *Forest plot of IE prevalence in EF-BSI stratified by median study enrolment year.*

Studies are grouped into three periods: before 2010 (blue), 2010–2019 (red), and 2020 and after (green). Before 2010: pooled 14·5% (95% CI 11·8–17·8%), I²=0·0%, k=2. 2010–2019: pooled 10·6% (95% CI 6·2–17·5%), I²=98·3%, τ²=0·8130, k=10. 2020 and after: pooled 12·8% (95% CI 6·7–22·9%), I²=82·7%, τ²=0·3118, k=3. Diamonds represent subgroup pooled estimates.

IE = infective endocarditis; EF-BSI = *Enterococcus faecalis* bloodstream infection.

**Supplementary Figure 5.** *Forest plot of IE prevalence in EF-BSI stratified by IE diagnostic criteria.*

Subgroup pooled estimates (diamonds) were generated for criteria with ≥2 contributing studies: Duke–ISCVID 2023 (dark red), pooled 19·7% (95% CI 15·0–25·3%), I²=76·2%, k=3; ICD-10 codes (purple), pooled 7·8% (95% CI 2·4–22·0%), I²=99·1%, k=2; modified Duke 2000 (light blue), pooled 14·9% (95% CI 11·3–19·5%), I²=86·0%, k=6. Fernandez-Guerrero 2002 (Duke 1994 criteria), Lindberg 2022 (ESC 2015), Consuegra Pérez (ESC 2023), and Nye 2024 (criteria not reported) each contributed a single study with no subgroup pooling.

IE = infective endocarditis; EF-BSI = *Enterococcus faecalis* bloodstream infection.

**Supplementary Figure 6.** *Forest plot of IE prevalence in EF-BSI stratified by Centre level.*

Studies are grouped into two groups: non-tertiary/mixed centers (blue) and tertiary centers (red). Non-tertiary/mixed: pooled 11·8% (95% CI 7·2–18·7%), I²=97·7%; tertiary: pooled 11·8% (95% CI 6·6–20·1%), I²=95·2%.

**Supplementary Figure 7.** *Forest plot of IE prevalence in EF-BSI excluding studies with high Risk of Bias.*

Pooled IE prevalence after exclusion of Nye 2024, Fernandez Guerrero 2002, Ostergaard 2019, Consuegra Pérez 2026, Lindberg 2022, Lai 2025, Wehage 2026, and including the studies with moderate or low Risk of Bias. Pooled prevalence: 17·0% (95% CI 13·6–21·1%), I²=87·6%*.*

**Supplementary Figure 8.** *Meta-regression of IE prevalence in EF-BSI as a function of echocardiographic coverage — sensitivity analysis substituting the modified Duke 2000 criteria for the Duke–ISCVID 2023 criteria in the Varisco et al (2026) study.*

Layout and conventions as in Figure 3. **(A)** TEE as moderator: quadratic model R²=83·2%, AIC=18·7 (vs 21·5 linear; ΔAIC=2·8; LRT p=0·027); residual I²=69·6%; estimated plateau 27·3% (95% CI 18·9–37·7%) at TEE coverage 64% (95% CI 40–88%). **(B)** TTE as moderator: quadratic model R²=68·5%, AIC=18·5 (vs 24·2 linear; ΔAIC=5·7; LRT p=0·006); residual I²=81·1%; estimated plateau 28·2% (95% CI 17·6–42·1%) at TTE coverage 73% (95% CI 64–83%).

IE = infective endocarditis; EF-BSI = *Enterococcus faecalis* bloodstream infection; TEE = transesophageal echocardiography; TTE = transthoracic echocardiography; AIC = Akaike information criterion; LRT = likelihood ratio test.

**Supplementary Figure 9.** *Forest plots of clinical predictors of IE in EF-BSI. Each panel shows the association between one clinical predictor and IE*.

Blue squares represent odds ratios (OR); red squares represent hazard ratios (HR), used where OR was not available. Adjusted estimates are labelled "(adj)"; unadjusted estimates are unlabelled. Diamonds represent random-effects pooled estimates, generated only where ≥2 studies reported the same effect measure for the same predictor. Horizontal lines show 95% confidence intervals. The reference line is drawn at the null value (OR/HR = 1·0).

BC = blood culture; EF-BSI = *Enterococcus faecalis* bloodstream infection; IE = infective endocarditis; OR = odds ratio; HR = hazard ratio.

**Supplementary Figure 10.** Funnel plot for assessment of publication bias. Each circle represents one study, plotted as logit-transformed observed IE prevalence (x-axis) against standard error (y-axis). The vertical solid line indicates the pooled estimate; dashed lines indicate the 95% pseudo-confidence region. Egger's test for funnel plot asymmetry: z = −0·948, p = 0·3602. The trim-and-fill method identified no missing studies (see main text). IE = infective endocarditis; EF-BSI = *Enterococcus faecalis* bloodstream infection.

**Supplementary Table 1**: Risk of bias assessment (QUIPS) of the included studies — domain-level judgements

| **Study** | **D1 Study participation** | **D2 Study attrition** | **D3 Prognostic factor measurement** | **D4 Outcome measurement** | **D5 Study confounding** | **D6 Statistical analysis and reporting** | **Overall RoB** | **Study** | **D1 Study participation** |
| --- | --- | --- | --- | --- | --- | --- | --- | --- | --- |
| Fernandez-Guerrero 2002 | Low | Moderate | Low | High | Moderate | Moderate | High | Fernandez-Guerrero 2002 | Low |
| Pinholt 2014 | Low | Low | Low | High | Moderate | Low | Moderate | Pinholt 2014 | Low |
| Dahl 2016 | Low | Low | Low | High | Moderate | Low | Moderate | Dahl 2016 | Low |
| Berge 2019 | Low | Low | Low | High | Moderate | Low | Moderate | Berge 2019 | Low |
| Dahl 2019 | Low | Low | Low | Low | Moderate | Low | Low | Dahl 2019 | Low |
| Ostergaard 2019 | Low | Low | Low | High | Moderate | Low | High | Ostergaard 2019 | Low |
| Escola Verge 2021 | Moderate | Low | Low | High | Moderate | Moderate | Moderate | Escola Verge 2021 | Moderate |
| Lindberg 2022 | Low | Low | Low | High | High | Moderate | High | Lindberg 2022 | Low |
| Nye 2024 | Moderate | High | Moderate | High | High | High | High | Nye 2024 | Moderate |
| Lai 2025 | Low | Moderate | Moderate | High | High | Moderate | High | Lai 2025 | Low |

Risk of bias was assessed with the Quality in Prognostic Studies (QUIPS) tool across six domains — study participation (D1), study attrition (D2), prognostic factor measurement (D3), outcome measurement (D4), study confounding (D5), and statistical analysis and reporting (D6). Each domain and the overall study were rated as low, moderate or high risk of bias. The overall rating reflects the number and the review-relevance of the domains at risk rather than a simple arithmetic combination: because the review outcome is IE prevalence, D4 carries the greatest weight, and studies were downgraded to Moderate rather than High when a High rating in D4 was offset by complete population-based ascertainment, independent adjudication or complete follow-up.. EF-BSI = *Enterococcus faecalis* bloodstream infection; IE = infective endocarditis; RoB = risk of bias.

**Supplementary Table 2**: Risk-of-bias sensitivity analysis — IE prevalence.

| **Analysis** | **k** | **EF-BSI, n** | **IE, n** | **Pooled prevalence, % (95% CI)** | **I², %** | **τ²** | **Δ vs primary, pp** | **Studies excluded** |
| --- | --- | --- | --- | --- | --- | --- | --- | --- |
| A. All studies (primary analysis) | 15 | 11157 | 1483 | 11.8 (8.1–16.8) | 97.3 | 0.595 | +0.0 | — |
| B. Excluding overall High risk of bias | 8 | 3413 | 573 | 17.0 (13.6–21.1) | 87.6 | 0.123 | +5.2 | Nye 2024; Fernandez Guerrero 2002; Ostergaard 2019 EHJ; Consuegra Pérez 2026; Lindberg 2022; Lai 2025 MEFIER; Wehage 2026 |
| C. Excluding echocardiographic coverage <50% | 4 | 1550 | 322 | 21.3 (16.8–26.5) | 79.9 | 0.067 | +9.5 | Nye 2024; Pinholt 2014; Fernandez Guerrero 2002; Ostergaard 2019 EHJ; Dahl 2016; Consuegra Pérez 2026; Lindberg 2022; Escola Verge 2021; Berge 2019; Lai 2025 MEFIER; Wehage 2026 |
| Stratum: Low risk of bias | 2 | 887 | 215 | 24.2 (21.5–27.2) | 0.0 | 0.0 | — | — |
| Stratum: Moderate risk of bias | 6 | 2526 | 358 | 14.7 (12.1–17.7) | 72.4 | 0.053 | — | — |
| Stratum: High risk of bias | 7 | 7744 | 910 | 6.9 (3.4–13.2) | 96.6 | 0.815 | — | — |

Sensitivity analysis of the pooled prevalence of infective endocarditis in Enterococcus faecalis bloodstream infection according to risk of bias. Risk of bias was assessed with the QUIPS tool. Pooled estimates were obtained with a binomial-normal random-effects model on logit-transformed proportions. Differences are expressed in percentage points relative to the primary analysis. CI, confidence interval; EF-BSI, Enterococcus faecalis bloodstream infection; IE, infective endocarditis; pp, percentage points.

**Supplementary Table 3**: Meta-regression coefficients — sensitivity analysis (Varisco: Modified Duke 2000)

| **Moderator** | **Model** | **Term** | **Estimate** | **SE** | **z** | **p_value** | **tau2** | **R2** | **AIC** |
| --- | --- | --- | --- | --- | --- | --- | --- | --- | --- |
| TEE coverage | Linear | Intercept | -2.58830 | 0.26042 | -9.939 | <0.001 | 0.14541 | 71.2% | 21.50 |
| TEE coverage | Linear | TEE proportion | 2.46263 | 0.74885 | 3.289 | 0.001 | 0.14541 | 71.2% | 21.50 |
| TEE coverage | Quadratic | Intercept | -3.19949 | 0.41604 | -7.690 | <0.001 | 0.08479 | 83.2% | 18.70 |
| TEE coverage | Quadratic | TEE proportion | 6.92115 | 2.46712 | 2.805 | 0.005 | 0.08479 | 83.2% | 18.70 |
| TEE coverage | Quadratic | TEE proportion^2^ | -5.39396 | 2.79113 | -1.933 | 0.053 | 0.08479 | 83.2% | 18.70 |
| TTE coverage | Linear | Intercept | -2.85863 | 0.40451 | -7.067 | <0.001 | 0.29499 | 41.5% | 24.20 |
| TTE coverage | Linear | TTE proportion | 1.67362 | 0.65832 | 2.542 | 0.011 | 0.29499 | 41.5% | 24.20 |
| TTE coverage | Quadratic | Intercept | -5.20346 | 0.98337 | -5.291 | <0.001 | 0.15875 | 68.5% | 18.50 |
| TTE coverage | Quadratic | TTE proportion | 11.64280 | 3.82286 | 3.046 | 0.002 | 0.15875 | 68.5% | 18.50 |
| TTE coverage | Quadratic | TTE proportion^2^ | -7.93703 | 2.95828 | -2.683 | 0.007 | 0.15875 | 68.5% | 18.50 |

Coefficients of the linear and quadratic meta-regression models of IE prevalence in EF-BSI on TEE and TTE echocardiographic coverage, in the sensitivity analysis substituting the modified Duke 2000 criteria for the Duke–ISCVID 2023 criteria in the Varisco et al (2026) study. Corresponds to Supplementary Figure 8. Coefficients and R² were estimated with the REML estimator; AIC values were obtained after refitting the models with the ML estimator, as REML-based models are not directly comparable on this criterion. AIC, Akaike information criterion; EF-BSI, *Enterococcus faecalis* bloodstream infection; IE, infective endocarditis; R², explained variance; SE, standard error; TEE, transesophageal echocardiography; TTE, transthoracic echocardiography; τ², between-study variance.

**Supplementary Table 4**: Leave-one-out sensitivity analysis

| **Study removed** | **Moderator** | **Model** | **R²** | **AIC** | **β₀ (intercept)** | **β₁** | **β₂** | **Plateau x** | **Plateau prev.** |
| --- | --- | --- | --- | --- | --- | --- | --- | --- | --- |
| Dahl_2019 | TEE coverage | Linear | 97.5% | 15.31 | -3.15548 | 6.02598 | NA | NA | NA |
| Nye_2024 | TEE coverage | Linear | 67.2% | 23.83 | -2.48753 | 2.44966 | NA | NA | NA |
| Dahl_2016 | TEE coverage | Linear | 67.3% | 24.20 | -2.59335 | 2.66107 | NA | NA | NA |
| Consuegra Pérez_2026 | TEE coverage | Linear | 68.4% | 16.36 | -2.26445 | 1.97634 | NA | NA | NA |
| Lindberg_2022 | TEE coverage | Linear | 73.0% | 19.66 | -2.38787 | 2.30202 | NA | NA | NA |
| Escola Verge_2021 | TEE coverage | Linear | 69.1% | 23.79 | -2.57807 | 2.54759 | NA | NA | NA |
| Berge_2019 | TEE coverage | Linear | 69.9% | 24.17 | -2.52595 | 2.55573 | NA | NA | NA |
| Robson_2025 | TEE coverage | Linear | 77.9% | 21.61 | -2.55575 | 2.45377 | NA | NA | NA |
| Varisco_2026 | TEE coverage | Linear | 81.8% | 22.05 | -2.51823 | 2.33155 | NA | NA | NA |
| Wehage_2026 | TEE coverage | Linear | 71.2% | 23.80 | -2.51888 | 2.53084 | NA | NA | NA |
| Dahl_2019 | TEE coverage | Quadratic | 97.5% | 16.93 | -2.95224 | 3.52549 | 6.293418 | NA | NA |
| Nye_2024 | TEE coverage | Quadratic | 98.3% | 11.77 | -4.11850 | 12.93631 | -11.862846 | 0.545 | 35.6% |
| Dahl_2016 | TEE coverage | Quadratic | 96.8% | 18.74 | -3.36532 | 8.85235 | -7.684345 | 0.576 | 30.7% |
| Consuegra Pérez_2026 | TEE coverage | Quadratic | 95.8% | 12.00 | -3.15100 | 7.82196 | -6.682594 | 0.585 | 29.7% |
| Lindberg_2022 | TEE coverage | Quadratic | 98.1% | 11.10 | -3.32752 | 8.85199 | -7.762576 | 0.570 | 30.9% |
| Escola Verge_2021 | TEE coverage | Quadratic | 99.0% | 18.06 | -3.44507 | 9.63801 | -8.602258 | 0.560 | 32.2% |
| Berge_2019 | TEE coverage | Quadratic | 100.0% | 15.13 | -3.28850 | 8.92097 | -7.936477 | 0.562 | 31.4% |
| Robson_2025 | TEE coverage | Quadratic | 98.1% | 15.82 | -3.32218 | 8.49074 | -7.278987 | 0.583 | 30.0% |
| Varisco_2026 | TEE coverage | Quadratic | 100.0% | 16.22 | -3.18425 | 7.48998 | -6.193096 | 0.605 | 28.5% |
| Wehage_2026 | TEE coverage | Quadratic | 98.2% | 16.97 | -3.32906 | 8.79480 | -7.679932 | 0.573 | 30.8% |
| Dahl_2019 | TTE coverage | Linear | 76.7% | 20.43 | -2.80276 | 1.87314 | NA | NA | NA |
| Nye_2024 | TTE coverage | Linear | 92.9% | 17.91 | -2.49247 | 1.46092 | NA | NA | NA |
| Dahl_2016 | TTE coverage | Linear | 82.0% | 20.29 | -2.84077 | 1.86717 | NA | NA | NA |
| Consuegra Pérez_2026 | TTE coverage | Linear | 87.7% | 11.77 | -2.45220 | 1.38874 | NA | NA | NA |
| Lindberg_2022 | TTE coverage | Linear | 88.1% | 16.62 | -2.55363 | 1.51879 | NA | NA | NA |
| Escola Verge_2021 | TTE coverage | Linear | 100.0% | 13.67 | -2.62029 | 1.51547 | NA | NA | NA |
| Berge_2019 | TTE coverage | Linear | 81.8% | 20.12 | -2.86311 | 1.89905 | NA | NA | NA |
| Robson_2025 | TTE coverage | Linear | 85.7% | 19.47 | -2.70504 | 1.64838 | NA | NA | NA |
| Varisco_2026 | TTE coverage | Linear | 83.1% | 19.66 | -2.83739 | 2.02818 | NA | NA | NA |
| Wehage_2026 | TTE coverage | Linear | 86.5% | 19.77 | -2.70724 | 1.71384 | NA | NA | NA |
| Dahl_2019 | TTE coverage | Quadratic | 93.6% | 15.09 | -4.56147 | 9.43774 | -6.130135 | 0.770 | 28.3% |
| Nye_2024 | TTE coverage | Quadratic | 98.7% | 9.88 | -4.33533 | 8.91617 | -5.732201 | 0.778 | 29.6% |
| Dahl_2016 | TTE coverage | Quadratic | 94.3% | 15.60 | -4.52225 | 9.22688 | -5.860408 | 0.787 | 29.1% |
| Consuegra Pérez_2026 | TTE coverage | Quadratic | 95.4% | 10.18 | -3.79357 | 6.69135 | -4.036011 | 0.829 | 26.5% |
| Lindberg_2022 | TTE coverage | Quadratic | 96.0% | 12.55 | -4.19812 | 8.13625 | -5.084671 | 0.800 | 28.0% |
| Escola Verge_2021 | TTE coverage | Quadratic | 100.0% | 9.91 | -3.96124 | 6.81175 | -3.992119 | 0.853 | 25.8% |
| Berge_2019 | TTE coverage | Quadratic | 94.6% | 13.76 | -5.00741 | 10.66869 | -6.820580 | 0.782 | 30.2% |
| Robson_2025 | TTE coverage | Quadratic | 96.9% | 13.22 | -5.27279 | 12.35556 | -8.213144 | 0.752 | 34.8% |
| Varisco_2026 | TTE coverage | Quadratic | 93.0% | 15.59 | -4.49230 | 9.01632 | -5.624110 | 0.802 | 29.3% |
| Wehage_2026 | TTE coverage | Quadratic | 96.7% | 14.07 | -4.39665 | 8.88932 | -5.644412 | 0.787 | 29.0% |

Leave-one-out sensitivity analysis of the meta-regression models of the primary analysis (Table 2, Figure 3; TEE and TTE coverage as moderators of IE prevalence in EF-BSI), refitting each model after omitting one study at a time. R², AIC, and the regression coefficients (β₀, β₁, β₂) are reported for each omitted study; plateau x and plateau prevalence give the coverage level and IE prevalence at the vertex of the quadratic curve, reported as NA where the quadratic term was not concave-down. Removal of Dahl et al [2] reversed the sign of the TEE quadratic coefficient, reflecting its position as the sole study with TEE coverage above the estimated vertex. AIC, Akaike information criterion; EF-BSI, Enterococcus faecalis bloodstream infection; IE, infective endocarditis; NA, not applicable; R², explained variance; TEE, transesophageal echocardiography; TTE, transthoracic echocardiography.

**Supplementary Table 5**: Influence analysis

| **Outcome** | **Study** | **Estimate, % (95% CI)** | **Weight, %** | **Pooled estimate omitting study, % (95% CI)** | **I² omitting study, %** |
| --- | --- | --- | --- | --- | --- |
| IE prevalence in EF-BSI | Dahl 2019 | 26.2 (21.8–31.1) | 7.3 | 11.6 (8.0–16.4) | 96.9 |
| IE prevalence in EF-BSI | Nye 2024 | 7.1 (3.9–12.8) | 6.3 | 12.7 (8.8–18.1) | 97.4 |
| IE prevalence in EF-BSI | Pinholt 2014 | 13.3 (10.5–16.8) | 7.3 | 12.1 (8.2–17.6) | 97.4 |
| IE prevalence in EF-BSI | Fernandez Guerrero 2002 | 21.6 (13.7–32.4) | 6.6 | 11.8 (8.0–16.9) | 97.4 |
| IE prevalence in EF-BSI | Ostergaard 2019 | 16.7 (15.7–17.8) | 7.5 | 11.9 (8.1–17.3) | 96.0 |
| IE prevalence in EF-BSI | Dahl 2016 | 12.1 (9.8–14.8) | 7.4 | 12.2 (8.3–17.7) | 97.3 |
| IE prevalence in EF-BSI | Consuegra Pérez 2026 | 1.4 (0.3–5.2) | 3.9 | 13.5 (9.8–18.2) | 96.5 |
| IE prevalence in EF-BSI | Lindberg 2022 | 3.2 (0.8–12.0) | 3.9 | 13.0 (9.1–18.1) | 97.1 |
| IE prevalence in EF-BSI | Escola Verge 2021 | 18.8 (15.1–23.1) | 7.3 | 11.8 (8.0–17.1) | 97.3 |
| IE prevalence in EF-BSI | Berge 2019 | 11.1 (8.4–14.6) | 7.2 | 12.3 (8.3–17.8) | 97.4 |
| IE prevalence in EF-BSI | Lai 2025 | 3.4 (2.8–4.2) | 7.4 | 14.2 (10.8–18.4) | 94.4 |
| IE prevalence in EF-BSI | Robson 2025 | 23.7 (16.8–32.3) | 6.9 | 11.7 (8.0–16.7) | 97.3 |
| IE prevalence in EF-BSI | Varisco 2026 | 23.0 (19.7–26.7) | 7.4 | 11.7 (8.0–16.7) | 96.9 |
| IE prevalence in EF-BSI | Zimmermann 2026 | 14.6 (11.9–17.8) | 7.4 | 12.1 (8.1–17.5) | 97.3 |
| IE prevalence in EF-BSI | Wehage 2026 | 10.6 (5.6–19.1) | 6.2 | 12.4 (8.4–17.8) | 97.5 |
| IE prevalence in EF-BSI | Pooled (all estimates) | 12.3 (8.6–17.3) | 100.0 | k = 15 | 97.1 |
| NOVA — sensitivity | Dahl 2016 | 97.4 (90.3–99.4) | 19.9 | 95.1 (84.7–98.6) | 54.7 |
| NOVA — sensitivity | Consuegra Pérez 2026 | 83.3 (19.4–99.0) | 8.7 | 96.2 (89.0–98.8) | 61.3 |
| NOVA — sensitivity | Lindberg 2022 | 83.3 (19.4–99.0) | 8.7 | 96.2 (89.0–98.8) | 61.3 |
| NOVA — sensitivity | Berge 2019 | 98.9 (84.6–99.9) | 9.8 | 94.9 (85.9–98.3) | 58.2 |
| NOVA — sensitivity | Berge 2019 — cohort 1 | 98.1 (76.4–99.9) | 9.7 | 95.2 (86.3–98.4) | 60.7 |
| NOVA — sensitivity | Varisco 2026 | 87.2 (80.1–92.0) | 28.4 | 97.4 (93.9–98.9) | 0.0 |
| NOVA — sensitivity | Zimmermann 2026 | 99.1 (94.1–99.9) | 14.8 | 94.0 (84.7–97.8) | 46.0 |
| NOVA — sensitivity | Pooled (all estimates) | 95.6 (88.3–98.4) | 100.0 | k = 7 | 56.1 |
| NOVA — specificity | Dahl 2016 | 23.5 (17.6–30.6) | 15.1 | 31.9 (20.9–45.4) | 95.8 |
| NOVA — specificity | Consuegra Pérez 2026 | 35.0 (17.7–57.4) | 10.5 | 30.0 (19.4–43.1) | 96.4 |
| NOVA — specificity | Lindberg 2022 | 8.3 (3.5–18.5) | 10.5 | 35.0 (27.2–43.6) | 90.7 |
| NOVA — specificity | Berge 2019 | 28.9 (24.4–33.8) | 15.9 | 30.7 (19.5–44.9) | 95.6 |
| NOVA — specificity | Berge 2019 — cohort 1 | 35.1 (29.4–41.3) | 15.7 | 29.6 (18.7–43.5) | 95.8 |
| NOVA — specificity | Varisco 2026 | 51.4 (46.6–56.2) | 16.1 | 27.8 (20.1–37.1) | 89.6 |
| NOVA — specificity | Zimmermann 2026 | 36.9 (32.5–41.5) | 16.1 | 29.3 (18.6–43.0) | 95.0 |
| NOVA — specificity | Pooled (all estimates) | 30.7 (21.3–42.1) | 100.0 | k = 7 | 94.9 |
| DENOVA — sensitivity | Consuegra Pérez 2026 | 83.3 (19.4–99.0) | 5.2 | 89.4 (78.7–95.0) | 59.6 |
| DENOVA — sensitivity | Lindberg 2022 | 83.3 (19.4–99.0) | 5.2 | 89.4 (78.7–95.0) | 59.6 |
| DENOVA — sensitivity | Berge 2019 | 98.9 (84.6–99.9) | 6.1 | 86.7 (76.9–92.7) | 43.5 |
| DENOVA — sensitivity | Berge 2019 — cohort 1 | 98.1 (76.4–99.9) | 6.0 | 87.1 (77.0–93.2) | 47.4 |
| DENOVA — sensitivity | Varisco 2026 | 79.2 (71.2–85.4) | 35.9 | 91.2 (85.7–94.7) | 0.0 |
| DENOVA — sensitivity | Zimmermann 2026 | 90.4 (83.5–94.6) | 31.8 | 89.4 (74.3–96.1) | 40.2 |
| DENOVA — sensitivity | Wehage 2026 | 87.5 (46.3–98.3) | 9.6 | 89.5 (77.9–95.3) | 61.8 |
| DENOVA — sensitivity | Pooled (all estimates) | 88.7 (78.9–94.3) | 100.0 | k = 7 | 50.6 |
| DENOVA — specificity | Consuegra Pérez 2026 | 85.0 (62.4–95.1) | 6.9 | 83.9 (77.0–89.0) | 87.8 |
| DENOVA — specificity | Lindberg 2022 | 76.7 (64.4–85.7) | 13.4 | 84.9 (78.5–89.7) | 85.9 |
| DENOVA — specificity | Berge 2019 | 83.0 (78.7–86.6) | 17.4 | 84.1 (76.4–89.6) | 85.1 |
| DENOVA — specificity | Berge 2019 — cohort 1 | 85.1 (80.1–89.1) | 16.6 | 83.7 (75.9–89.3) | 86.6 |
| DENOVA — specificity | Varisco 2026 | 83.0 (79.1–86.3) | 17.7 | 84.1 (76.4–89.6) | 84.5 |
| DENOVA — specificity | Zimmermann 2026 | 93.1 (90.3–95.1) | 16.4 | 82.7 (80.3–84.8) | 0.0 |
| DENOVA — specificity | Wehage 2026 | 71.4 (54.6–83.9) | 11.7 | 85.3 (79.5–89.6) | 83.8 |
| DENOVA — specificity | Pooled (all estimates) | 84.0 (77.8–88.7) | 100.0 | k = 7 | 84.4 |

Leave-one-out influence analysis of the pooled prevalence of IE in EF-BSI (GLMM, as in the primary analysis) and of the pooled sensitivity and specificity of the NOVA and DENOVA scores (random-effects model on logit-transformed proportions, REML), refitted after omitting one study or one score estimate at a time. For each outcome, the observed estimate and weight of the omitted study are shown alongside the recalculated pooled estimate and I². CI, confidence interval; EF-BSI, *Enterococcus faecalis* bloodstream infection; GLMM, generalised linear mixed model; I², between-study heterogeneity statistic; IE, infective endocarditis.

**Supplementary Table 6**: Risk-of-bias sensitivity analysis — prediction scores

| **Score** | **Metric** | **k (all)** | **All studies, % (95% CI)** | **I² all, %** | **k (High RoB excluded)** | **High RoB excluded, % (95% CI)** | **I² restricted, %** | **Studies excluded** |
| --- | --- | --- | --- | --- | --- | --- | --- | --- |
| NOVA | Sensitivity | 6 | 95.6 (88.3–98.4) | 56.1 | 4 | 96.9 (89.8–99.1) | 67.2 | Consuegra Pérez 2026; Lindberg 2022 |
| NOVA | Specificity | 6 | 30.7 (21.3–42.1) | 94.9 | 4 | 34.9 (26.4–44.6) | 93.2 | Consuegra Pérez 2026; Lindberg 2022 |
| DENOVA | Sensitivity | 6 | 88.7 (78.9–94.3) | 50.6 | 3 | 92.0 (76.3–97.6) | 84.4 | Consuegra Pérez 2026; Lindberg 2022; Wehage 2026 |
| DENOVA | Specificity | 6 | 84.0 (77.8–88.7) | 84.4 | 3 | 86.6 (80.2–91.2) | 89.0 | Consuegra Pérez 2026; Lindberg 2022; Wehage 2026 |
| MEFIER | Sensitivity | 2 | 54.5 (24.9–81.3) | 84.6 | 1 | Not estimable | — | Lai 2025 MEFIER |
| MEFIER | Specificity | 2 | 81.0 (66.2–90.2) | 95.1 | 1 | Not estimable | — | Lai 2025 MEFIER |

Pooled sensitivity and specificity of the NOVA, DENOVA and MEFIER scores before and after exclusion of studies rated High risk of bias. Proportions were pooled with an inverse-variance random-effects model on logit-transformed proportions (REML). Where fewer than two estimates remained after exclusion, no pooled estimate was calculated, and the result is reported as not estimable. Two-by-two tables not reported by the original authors were reconstructed by applying the reported sensitivity and specificity to the corresponding marginals. The increase in heterogeneity for pooled DENOVA sensitivity after exclusion reflects the removal of two estimates reporting 100% sensitivity based on two endocarditis cases each; these estimates were effectively uninformative and had masked the contrast between derivation-cohort performance and external validation. CI, confidence interval; RoB, risk of bias.

**Studies excluded after full-text assessment** (n = 44)

| **Citation** | **Reason for exclusion** |
| --- | --- |
| Bayless DR, Baddour LM, Lahr BD, Hagan GA, Cao JJ, DeSimone DC. A 5-Year Epidemiological Study of Monomicrobial Enterococcal Bloodstream Infection in a Population-Based Cohort. Open Forum Infect Dis. 2025;12(9):ofaf506. | Mixed population / data not separable by species |
| Munita JM, Contreras G, Sahasrabhojane P, Sanchez Petitto G, Araos R, Wanger A, Aitken SL, Shelburne SA, Arias CA. Abstract 1890. A Prospective Study of Enterococcal Bacteremia in Cancer vs. Non-Cancer Populations: One Disease, Two Tales. Open Forum Infect Dis. 2017;4(Suppl 1):S546. IDWeek 2017. | Conference abstract |
| Lwin N, Bannan A. A retrospective observational study on enterococcal bacteraemia and endocarditis at a regional hospital in New South Wales, Australia. Infect Dis Health. 2020;25(4):245-252. | Mixed population / data not separable by species |
| Araoka H, Kimura M, Yoneyama A. A surveillance of high-level gentamicin-resistant enterococcal bacteremia. J Infect Chemother. 2011;17(3):433-434. | Mixed population / data not separable by species |
| Try Lenz I, Pries-Heje M, Hjulmand J, Hasselbalch RB, Jarløv JO, Faurholt-Jepsen D, Moser C, Iversen K, Bundgaard H. Characteristics and outcomes in patients with infective endocarditis caused by Enterococcus faecium and Enterococcus faecalis. Eur Heart J. 2022;43(Suppl 2):ehac544.1669. ESC Congress 2022, Barcelona. | Conference abstract |
| Lupia T, Roberto G, Scaglione L, Shbaklo N, De Benedetto I, Scabini S, Mornese Pinna S, Curtoni A, Cavallo R, De Rosa FG, Corcione S. Clinical and microbiological characteristics of bloodstream infections caused by Enterococcus spp. within internal medicine wards: a two-year single-centre experience. Intern Emerg Med. 2022;17(4):1129-1137. | Mixed population / data not separable by species |
| Alkan-Çeviker S, Günal Ö, Köksal E, Aygün C, Kılıç SS. Comparison of Health Care-Associated Enterococcus faecium and Enterococcus faecalis Bloodstream Infections. Klimik Derg. 2020;33(1):87-90. | Mixed population / data not separable by species |
| Mattar C, Thomas BS, Strasserking F, Bakeer F, Chung J, Warren D. Abstract 1898. Development and Validation of a Risk Prediction Score For Enterococcal Endocarditis. Open Forum Infect Dis. 2017;4(Suppl 1). IDWeek 2017. | Conference abstract |
| Cabiltes I, Coghill S, Bowe SJ, Athan E. Enterococcal bacteraemia 'silent but deadly': a population-based cohort study. Intern Med J. 2020;50(2):e14396. | Mixed population / data not separable by species |
| Berge A, Årdal L, Nilson B, Rasmussen M. Enterococcus faecalis bacteremia, cardiac implantable electronic device, extraction, and the risk of recurrence. Infection. 2022;50(6):1517-1523. | Incompatible design |
| Cattaneo C, Rieg S, Schwarzer G, Müller MC, Blümel B, Kern WV. Enterococcus faecalis bloodstream infection: does infectious disease specialist consultation make a difference? Infection. 2021;49(6):1289-1297. | Mixed population / data not separable by species |
| Alkan S, Kuloğlu F, Akata F. Enterokok Bakteriyemilerinde Risk Faktörlerinin Değerlendirilmesi [Evaluation of Risk Factors in Enterococcal Bloodstream Infections]. Klimik Derg. 2016;29(2):65-70. | Mixed population / data not separable by species |
| De Oliveira EK, Van Vaerenbergh K, Shumkova M, et al. Epidemiology of bloodstream infections and infective endocarditis: new insights on typically associated species definitions and use of echocardiography screening. Eur Heart J. 2025;46(Suppl 1):ehaf784.2506. ESC Congress 2025. | Conference abstract |
| Al Abri M, Ba Wazir H. Epidemiology, Clinical and Microbiological Characteristics, and Outcomes of Enterococcus Bacteraemia: A 5-year experience of a university hospital in Oman. Sultan Qaboos Univ Med J. 2025;25:737-745. | Mixed population / data not separable by species |
| Fourré N, Zimmermann V, Ianculescu N, et al. Evaluating Blood Culture Parameters to Identify Patients at Low Risk of Infective Endocarditis Among Those With Bacteremia by Gram-positive Cocci. Open Forum Infect Dis. 2025;12(9):ofaf518. | Mixed population / data not separable by species |
| Zimmermann V, Fourré N, Ledergerber B, Epprecht J, Siedentop B, Monney P, Tzimas G, Frank M, Senn L, Ianculescu N, Niclauss L, Kirsch M, Van Hemelrijck M, Dzemali O, Guery B, Hasse B, Papadimitriou-Olivgeris M. Evaluation of Different Versions of the Duke Criteria for the Diagnosis of Infective Endocarditis Among Patients With Enterococcal Bacteremia; a Multicenter Study. Open Forum Infect Dis. 2025;12(7):ofaf408. | Mixed population / data not separable by species |
| Powers CE, Bookstaver PB, Caulder C, Bouknight A, Justo JA, Kohn J, Winders HR, Al-Hasan MN. Evaluation of early clinical failure criteria in Enterococcus species bloodstream infection. Infection. 2022;50(4):873-877. | Mixed population / data not separable by species |
| Pinholt M, Andersen CØ, Arpi M, Knudsen J. Abstract P922. Focus and outcome for community-acquired Enterococcus faecalis bacteraemia. Clin Microbiol Infect. 2011;17(Suppl 4):S230. ECCMID 2011. | Conference abstract |
| Foo H, Chater M, Maley M, van Hal SJ. Glycopeptide use is associated with increased mortality in Enterococcus faecalis bacteraemia. J Antimicrob Chemother. 2014;69(8):2252-2257. | Incompatible design |
| Lopes D, Grandbastien B, Orasch C, Greub G, Croxatto A, Prod'Hom G, Guery B. Impact of rapid identification by MALDI-TOF MS from positive blood cultures in Enterococcus spp. bloodstream infections. Eur J Clin Microbiol Infect Dis. 2025;44(5):1185-1196. | Mixed population / data not separable by species |
| Bartoletti M, Tedeschi S, Scudeller L, et al. Impact on Mortality of a Bundle for the Management of Enterococcal Bloodstream Infection. Open Forum Infect Dis. 2019;6(12):ofz473. | Mixed population / data not separable by species |
| Hedvat J, Gitman MR, Nwoye A, Chen L, Martin C, Wang M, Vasa C, Cusumano JA. Abstract 184. Implications of Enterococcus faecalis penicillin susceptibility in patients with bacteremia. Open Forum Infect Dis. 2023;10(Suppl 2):ofad500.257. IDWeek 2023. | Conference abstract |
| Papamanoli A, Yoo J, Mojahedi A, Jacob R, Grewal P, Dhaliwal S, Coritsidis A, Nakamura J, Fung J, Hotelling J, Rawal S, Karkala N, Tsui ST, Abata J, Psevdos G, Kalogeropoulos A, Marcos L. Abstract 53. Incidence of Bloodstream Infections and Outcomes in Patients with Severe COVID-19 Pneumonia. Open Forum Infect Dis. 2020;7(Suppl 1). IDWeek 2020. | Conference abstract |
| Lee RA, Vo DT, Zurko JC, Griffin RL, Rodriguez JM, Camins BC. Infectious Diseases Consultation Is Associated With Decreased Mortality in Enterococcal Bloodstream Infections. Open Forum Infect Dis. 2020;7(3):ofaa064. | Mixed population / data not separable by species |
| Ostovar R, Necaev AM, Schröter F, Seifi Zinab F, Dörr G, Schmalz G, Albes JM. Infectious Endocarditis Is Associated with Dental Treatment or Poor Dental Status-Results from the Brandenburg Endocarditis Registry (B.E.R.). J Clin Med. 2025;14(8):2784. | Incompatible design |
| Scolarici MJ, Berman LR, Saddler C, Smith J. Abstract 2113. Infectious endocarditis in patients with hematologic malignancy at the University of Wisconsin. Open Forum Infect Dis. 2022;9(Suppl 2):ofac492.1734. IDWeek 2022. | Conference abstract |
| Engers D, Siddiqui MN, Abunayala A, Jagarlamudi R, Infective Endocarditis in Enterococcus faecalis Bloodstream Infection:  Prevalence, Risk Factors, and Patient Outcomes. Open Forum Infect Dis. 2022;9(Suppl 2). IDWeek 2022. | Conference abstract |
| Freling SR, Richie I, Norwitz D, Canamar CP, Banerjee J, Davar K, Clark D, Spellberg B. Interpreting Blood Culture Results as Early Guidance for Infective Endocarditis. JAMA Netw Open. 2025;8(5):e258079. | Incompatible design |
| Gawryszewska I, Żabicka D, Bojarska K, Malinowska K, Hryniewicz W, Sadowy E. Invasive enterococcal infections in Poland: the current epidemiological situation. Eur J Clin Microbiol Infect Dis. 2016;35(5):847-856. | Mixed population / data not separable by species |
| Sunnerhagen T, Rasmussen M. Lower Specificity of the European Society of Cardiology 2023 Diagnostic Criteria for Infective Endocarditis When Spondylodiscitis Is Regarded as a Vascular Phenomenon. Clin Infect Dis. 2024;79(3):612-614. | Incompatible design |
| Casapao AM, Kullar R, Davis SL, et al. Multicenter Study of High-Dose Daptomycin for Treatment of Enterococcal Infections. Antimicrob Agents Chemother. 2013;57(9):4278-4286. | Mixed population / data not separable by species |
| Lindberg H, Berge A, Jovanovic-Stjernqvist M, et al. Performance of the 2023 Duke-International Society of Cardiovascular Infectious Diseases Diagnostic Criteria for Infective Endocarditis in Relation to the Modified Duke Criteria and to Clinical Management-Reanalysis of Retrospective Bacteremia Cohorts. Clin Infect Dis. 2024;78(4):956-963. | Incompatible design |
| Zhou X, Tang G. Prevalence of Infective Endocarditis in Enterococcus faecalis Bacteremia, Methodology Issues. J Am Coll Cardiol. 2019;74(19):2432-2433. | Incompatible design |
| De Oliveira EK, Van Vaerenbergh K, Shumkova M, et al. Prevalence of infective endocarditis in patients with bloodstream infections and deployment of echocardiographic resources in a single-center registry. Eur Heart J Cardiovasc Imaging. 2025;26(Suppl 1):jeae333.139. | Conference abstract |
| Zerbato V, Pol R, Sanson G, Suru DA, Pin E, Tabolli V, Monticelli J, Busetti M, Toc DA, Crocè LS, Luzzati R, Di Bella S. Risk Factors for 30-Day Mortality in Nosocomial Enterococcal Bloodstream Infections. Antibiotics (Basel). 2024;13(7):601. | Mixed population / data not separable by species |
| Andersen MH, Holle SLK, Klein CF, Bruun NE, Arpi M, Bundgaard H, Tønder N, Iversen KK. Risk for infective endocarditis in bacteremia with Gram positive cocci. Infection. 2020;48(6):905-912. | Cohort overlap |
| Oldberg K, Thorén R, Nilson B, Gilje P, Inghammar M, Rasmussen M. Short time to blood culture positivity in Enterococcus faecalis infective endocarditis. Eur J Clin Microbiol Infect Dis. 2021;40(8):1657-1664. | Cohort overlap |
| Dahl A, Fowler VG, Miro JM, Bruun NE. Sign of the Times: Updating Infective Endocarditis Diagnostic Criteria to Recognize Enterococcus faecalis as a Typical Endocarditis Bacterium. Clin Infect Dis. 2022;75(6):1097-1102. | Incompatible design |
| Bouza E, Kestler M, Beca T, Mariscal G, Rodríguez-Créixems M, Bermejo J, Fernández-Cruz A, Fernández-Avilés F, Muñoz P; Grupo de Apoyo al Manejo de la Endocarditis. The NOVA Score: A Proposal to Reduce the Need for Transesophageal Echocardiography in Patients With Enterococcal Bacteremia. Clin Infect Dis. 2015;60(4):528-535. | Mixed population / data not separable by species |
| Pérez-Rodríguez MT, Lima O, Garrido M, et al. The role of time to positive blood cultures in enhancing the predictive capability of DENOVA score for diagnosing infective endocarditis in patients with Enterococcus faecalis bacteremia. Eur J Clin Microbiol Infect Dis. 2024;43(7):1481-1486. | Incompatible design |
| Marcus JE, Ford MB, Sattler LA, Iqbal S, Garner CL, Sobieszczyk MJ, Barsoumian AE. Treatment and outcome of gram-positive bacteremia in patients receiving extracorporeal membrane oxygenation. Heart Lung. 2023;60:15-19. | Mixed population / data not separable by species |
| Fosbøl EL, Stahl A, Røder A, Nordsten CB, Østergaard L, Sehested TSG, Dahl A, Ihlemann N, Bundgaard H, Iversen K, Valeur N, Gislason G, Torp-Pedersen C, Voldstedlund M, Bagi P, Køber L. Transurethral resection of the prostate (TUR-P) and associated risk of infective endocarditis. Infection. 2025;53(4):1351-1361. | Cohort overlap |
| Tellapragada C, Östlund H, Giske C, Rasmussen M, Berge A. Recurrent bacteremia with Enterococcus faecalis, the clinical findings predicting endocarditis, and genomic characterization of the isolates: a retrospective cohort study. Eur J Clin Microbiol Infect Dis. 2023;42(8):1001-1009. | Incompatible design |
| Zimmermann V, Fourré N, Senn L, Guery B, Papadimitriou-Olivgeris M. Predictors of mortality of enterococcal bacteraemia and the role of source control interventions; a retrospective cohort study. Infection. 2025;53(5):2149-2158. | Mixed population / data not separable by species |

Studies identified through the search strategy that met full-text review but were excluded from the systematic review and meta-analysis, with the reason for exclusion for each. Reasons for exclusion were: mixed bacterial population in which EF-BSI episodes or EF-IE cases could not be separated by species; conference abstract; incompatible study design (e.g., case-control design or an IE-enriched denominator); and cohort overlap with an already-included study. EF-BSI, Enterococcus faecalis bloodstream infection; IE, infective endocarditis.

**PRISMA 2020 CHECKLIST**

*PRISMA 2020 checklist for this systematic review and meta-analysis.*

| **Section and Topic** | **Item #** | **Checklist item** | **Location where item is reported** |
| --- | --- | --- | --- |
| **TITLE** | | |  |
| Title | 1 | Identify the report as a systematic review. | Manuscript, p.1 (title identifies the work as “a Systematic Review and Meta-Analysis”). |
| **ABSTRACT** | | |  |
| Abstract | 2 | See the PRISMA 2020 for Abstracts checklist. | Manuscript, p.2 (structured Abstract: Background, Methods, Findings, Interpretation, Funding). |
| **INTRODUCTION** | | |  |
| Rationale | 3 | Describe the rationale for the review in the context of existing knowledge. | Manuscript, p.5 (Introduction, paragraph 1). |
| Objectives | 4 | Provide an explicit statement of the objective(s) or question(s) the review addresses. | Manuscript, p.6 (Introduction, closing paragraph, before Methods). |
| **METHODS** | | |  |
| Eligibility criteria | 5 | Specify the inclusion and exclusion criteria for the review and how studies were grouped for the syntheses. | Manuscript, p.6 (Methods – Eligibility criteria); Supplementary Methods, p.2 (Eligibility criteria) for full detail on exclusions and handling of non-standard criteria/echocardiography. |
| Information sources | 6 | Specify all databases, registers, websites, organisations, reference lists and other sources searched or consulted to identify studies. Specify the date when each source was last searched or consulted. | Manuscript, p.6 (Methods – Literature search and data extraction: 4 databases, primary search to 20 Jan 2026, re-run 16 Aug 2026); Supplementary Methods, p.2 (Literature search) for per-database detail. |
| Search strategy | 7 | Present the full search strategies for all databases, registers and websites, including any filters and limits used. | Supplementary Methods, p.2 (Literature search – full PubMed search string and equivalent strategies for other databases; reference-list and personal-file screening). |
| Selection process | 8 | Specify the methods used to decide whether a study met the inclusion criteria of the review, including how many reviewers screened each record and each report retrieved, whether they worked independently, and if applicable, details of automation tools used in the process. | Manuscript, pp.6–7 (Methods – Rayyan, two independent reviewers G.M./N.S., disagreements resolved by Al.Ca.); Supplementary Methods, pp.2–3 (Literature search / same detail). |
| Data collection process | 9 | Specify the methods used to collect data from reports, including how many reviewers collected data from each report, whether they worked independently, any processes for obtaining or confirming data from study investigators, and if applicable, details of automation tools used in the process. | Manuscript, p.7 (Methods: “Data were extracted on study characteristics, outcomes, IE predictors, prediction score performance, and echocardiographic strategy”); Supplementary Methods, p.3 (Data extraction: N.S. extracted, G.M. and An.Co. independently checked/verified); Author Contributions, Manuscript p.20 (confirms same process). |
| Data items | 10a | List and define all outcomes for which data were sought. Specify whether all results that were compatible with each outcome domain in each study were sought (e.g. for all measures, time points, analyses), and if not, the methods used to decide which results to collect. | Supplementary Methods, p.2 (Eligibility criteria: primary outcome is IE prevalence in EF-BSI, regardless of diagnostic criteria used); Manuscript, p.6 (Eligibility criteria) for the equivalent statement. |
|  | 10b | List and define all other variables for which data were sought (e.g. participant and intervention characteristics, funding sources). Describe any assumptions made about any missing or unclear information. | Supplementary Methods, p.3 (Data extraction – domains: study characteristics; population and outcomes; IE predictors and effect estimates; prediction scores and diagnostic performance; risk of bias; subgroup data), and p.3 (Wilson score method used to impute missing 95% CIs). |
| Study risk of bias assessment | 11 | Specify the methods used to assess risk of bias in the included studies, including details of the tool(s) used, how many reviewers assessed each study and whether they worked independently, and if applicable, details of automation tools used in the process. | Manuscript, p.7 (Methods – QUIPS tool cited); Supplementary Methods, p.3 (Risk of bias assessment – six QUIPS domains, N.S./G.M. independent assessment, Al.Ca. resolves conflicts); domain definitions also in Table 1 footnote, Manuscript p.26, and Supplementary Table 1, p.8. |
| Effect measures | 12 | Specify for each outcome the effect measure(s) (e.g. risk ratio, mean difference) used in the synthesis or presentation of results. | Manuscript, pp.7–8 (Methods – Statistical analysis: prevalence on logit scale via GLMM; sensitivity/specificity/AUROC for prediction scores); Supplementary Methods, pp.3–4 (Statistical analysis). |
| Synthesis methods | 13a | Describe the processes used to decide which studies were eligible for each synthesis (e.g. tabulating the study intervention characteristics and comparing against the planned groups for each synthesis (item #5)). | Manuscript, p.6 (Eligibility criteria – studies grouped/stratified rather than excluded for heterogeneous echocardiography or diagnostic criteria); Supplementary Methods, p.5 (main analysis stratified by echocardiographic strategy, region, period, diagnostic criteria, centre level, RoB). |
|  | 13b | Describe any methods required to prepare the data for presentation or synthesis, such as handling of missing summary statistics, or data conversions. | Supplementary Methods, p.3 (Wilson score method for missing 95% CIs) and p.4 (delta-method within-study variances for logit-transformed prevalences used in meta-regression). |
|  | 13c | Describe any methods used to tabulate or visually display results of individual studies and syntheses. | Manuscript – Tables 1–4 (pp.26–29) and Figures 1–4 (pp.25); Supplementary Figures 1–10 (pp.6–7) and Supplementary Tables 1–6 (pp.8–13). |
|  | 13d | Describe any methods used to synthesize results and provide a rationale for the choice(s). If meta-analysis was performed, describe the model(s), method(s) to identify the presence and extent of statistical heterogeneity, and software package(s) used. | Manuscript, p.7 (Methods – Statistical analysis: GLMM with logit link, ML estimation, REML for meta-regression coefficients/R², ML for AIC/LRT model comparison); Supplementary Methods, pp.3–4 (model specification) and p.5 (software: R 4.2.3, metafor, ggplot2, patchwork, dplyr, tidyr, scales, ggrepel, officer, DiagrammeR). |
|  | 13e | Describe any methods used to explore possible causes of heterogeneity among study results (e.g. subgroup analysis, meta-regression). | Manuscript, p.7 (Methods – meta-regression with TEE/TTE coverage as moderators; stratified subgroup analyses); Supplementary Methods, pp.4–5 (meta-regression and stratification methods in full). |
|  | 13f | Describe any sensitivity analyses conducted to assess robustness of the synthesized results. | Manuscript, p.7 (Methods – leave-one-out analyses; repetition of analyses substituting modified Duke 2000 for Duke–ISCVID 2023 in Varisco et al.); Supplementary Methods, p.4 (LOO sensitivity analysis and Duke-criteria substitution). |
| Reporting bias assessment | 14 | Describe any methods used to assess risk of bias due to missing results in a synthesis (arising from reporting biases). | Manuscript, p.8 (Methods – Publication bias: funnel plot, Egger’s test, trim-and-fill); Supplementary Methods, p.5 (Publication bias). |
| Certainty assessment | 15 | Describe any methods used to assess certainty (or confidence) in the body of evidence for an outcome. | Manuscript, p.8 (Methods – GRADE across risk of bias, inconsistency, indirectness, imprecision, publication bias); Supplementary Methods, p.5 (Evidence strength). |
| **RESULTS** | | |  |
| Study selection | 16a | Describe the results of the search and selection process, from the number of records identified in the search to the number of studies included in the review, ideally using a flow diagram. | Manuscript, p.8 (Results – Studies’ selection: 2265 records retrieved, 1274 screened, 57 assessed at full text ⬚ 2 more identified, 15 studies included); Figure 1 (PRISMA flow diagram), Manuscript p.25/legend. |
|  | 16b | Cite studies that might appear to meet the inclusion criteria, but which were excluded, and explain why they were excluded. | Manuscript, p.8 (Results – Studies’ selection: of 59 full-text reports, 44 excluded – 21 mixed/non-separable populations, 10 conference abstracts, 10 incompatible designs, 3 overlapping cohorts). Full citation-level list with individual reason for exclusion in Supplementary Material, “Studies excluded after full-text assessment” table, pp.14–16. |
| Study characteristics | 17 | Cite each included study and present its characteristics. | Manuscript, pp.8–9 (Results – Characteristics of the included studies and RoB assessment) and Table 1, p.26 (per-study country, design, enrolment period, N EF-BSI, N IE, IE criteria, echo strategy). |
| Risk of bias in studies | 18 | Present assessments of risk of bias for each included study. | Manuscript, p.9 (Results: “Overall risk of bias was low in two studies, moderate in six, and high in seven”) and Table 1 footnote, p.26; full per-domain judgements in Supplementary Table 1, p.8. |
| Results of individual studies | 19 | For all outcomes, present, for each study: (a) summary statistics for each group (where appropriate) and (b) an effect estimate and its precision (e.g. confidence/credible interval), ideally using structured tables or plots. | Table 1, Manuscript p.26 (per-study IE prevalence with 95% CI); Table 3, Manuscript p.28 (per-study sensitivity/specificity of NOVA, DENOVA, MEFIER with 95% CI). |
| Results of syntheses | 20a | For each synthesis, briefly summarise the characteristics and risk of bias among contributing studies. | Manuscript, pp.8–9 (Results – Characteristics of the included studies and RoB assessment, preceding the pooled-prevalence synthesis). |
|  | 20b | Present results of all statistical syntheses conducted. If meta-analysis was done, present for each the summary estimate and its precision (e.g. confidence/credible interval) and measures of statistical heterogeneity. If comparing groups, describe the direction of the effect. | Manuscript, pp.9–12 (Results – Pooled prevalence of IE in EF-BSI; Meta-regression; Prediction scores diagnostic performance) and Tables 2–4, pp.27–29. |
|  | 20c | Present results of all investigations of possible causes of heterogeneity among study results. | Manuscript, pp.9–11 (Results – stratified analyses by echo strategy/region/period/criteria/centre level/RoB, and Meta-regression by TEE/TTE coverage); Supplementary Figures 2–6, pp.6–7, and Supplementary Table 2, p.8. |
|  | 20d | Present results of all sensitivity analyses conducted to assess the robustness of the synthesized results. | Manuscript, pp.9–12 (sensitivity analyses reported alongside each result: Duke 2000 substitution, high-RoB exclusion, low-coverage exclusion, LOO for meta-regression and for prediction-score pooling); Supplementary Figures 1, 7, 8 and Supplementary Tables 2–4 and 6, pp.6–13. |
| Reporting biases | 21 | Present assessments of risk of bias due to missing results (arising from reporting biases) for each synthesis assessed. | Manuscript, p.12 (Results – Publication bias: funnel plot, Egger’s test p=0·36, trim-and-fill); Supplementary Figure 10, p.7 (Egger’s z=−0·948, p=0·3602). |
| Certainty of evidence | 22 | Present assessments of certainty (or confidence) in the body of evidence for each outcome assessed. | Manuscript, p.12 (Results – Certainty of evidence) and Table 4, p.29 (GRADE summary of findings). |
| **DISCUSSION** | | |  |
| Discussion | 23a | Provide a general interpretation of the results in the context of other evidence. | Manuscript, pp.13–16 (Discussion, paragraphs 1–5). |
|  | 23b | Discuss any limitations of the evidence included in the review. | Manuscript, pp.17–18 (Study limitations paragraph). |
|  | 23c | Discuss any limitations of the review processes used. | Manuscript, p.18 (Study limitations: underpowered Egger’s test, no individual patient data, sex-disaggregated data not poolable, no cost-effectiveness/RCT evidence). |
|  | 23d | Discuss implications of the results for practice, policy, and future research. | Manuscript, pp.14–19 (Discussion, throughout, and Conclusions, pp.18–19: clinical/practice implications, guideline implications, and research priorities). |
| **OTHER INFORMATION** | | |  |
| Registration and protocol | 24a | Provide registration information for the review, including register name and registration number, or state that the review was not registered. | Manuscript, p.6 (Methods: “prospectively registered in the International Prospective Register of Systematic Reviews (PROSPERO; CRD420251240242)”) and p.20 (Data Availability: “prospectively registered in PROSPERO on 3 April 2026 (registration number CRD420251240242)”). |
|  | 24b | Indicate where the review protocol can be accessed, or state that a protocol was not prepared. | Manuscript, p.6 (Methods, first paragraph: “prospectively registered in the International Prospective Register of Systematic Reviews (PROSPERO; CRD420251240242)”) – the protocol is accessible via the PROSPERO registration under this number. |
|  | 24c | Describe and explain any amendments to information provided at registration or in the protocol. | NA |
| Support | 25 | Describe sources of financial or non-financial support for the review, and the role of the funders or sponsors in the review. | Manuscript, p.2 (Abstract – Funding: “supported by internal fundings”) and p.20 (Financial report: “supported by internal funding”; Role of the funding source paragraph, p.8: no external funding, corresponding author had full data access and final responsibility for submission). |
| Competing interests | 26 | Declare any competing interests of review authors. | Manuscript, p.20 (Declaration of interests – Transparency Declaration: “authors declare no conflicts of interest related to this study”). |
| Availability of data, code and other materials | 27 | Report which of the following are publicly available and where they can be found: template data collection forms; data extracted from included studies; data used for all analyses; analytic code; any other materials used in the review. | NA |

From: Page MJ, McKenzie JE, Bossuyt PM, Boutron I, Hoffmann TC, Mulrow CD, et al. The PRISMA 2020 statement: an updated guideline for reporting systematic reviews. BMJ 2021;372:n71. doi: 10.1136/bmj.n71. This work is licensed under CC BY 4.0. To view a copy of this license, visit https://creativecommons.org/licenses/by/4.0/
